# Genetic Dissection of an Exogenously Induced Biofilm in Laboratory and Clinical Isolates of *E. coli*

**DOI:** 10.1371/journal.ppat.1000432

**Published:** 2009-05-15

**Authors:** Sasan Amini, Hani Goodarzi, Saeed Tavazoie

**Affiliations:** Department of Molecular Biology and Lewis-Sigler Institute for Integrative Genomics, Princeton University, Princeton, New Jersey, United States of America; University College Cork, Ireland

## Abstract

Microbial biofilms are a dominant feature of many human infections. However, developing effective strategies for controlling biofilms requires an understanding of the underlying biology well beyond what currently exists. Using a novel strategy, we have induced formation of a robust biofilm in *Escherichia coli* by utilizing an exogenous source of poly-*N*-acetylglucosamine (PNAG) polymer, a major virulence factor of many pathogens. Through microarray profiling of competitive selections, carried out in both transposon insertion and over-expression libraries, we have revealed the genetic basis of PNAG-based biofilm formation. Our observations reveal the dominance of electrostatic interactions between PNAG and surface structures such as lipopolysaccharides. We show that regulatory modulation of these surface structures has significant impact on biofilm formation behavior of the cell. Furthermore, the majority of clinical isolates which produced PNAG also showed the capacity to respond to the exogenously produced version of the polymer.

## Author Summary

Both in the wild and in the clinical setting many bacterial species live within surface-attached communities called biofilms. It is still unclear the extent to which the biofilm lifestyle and its associated phenotypes, such as hyper-tolerance to antimicrobial agents, can be attributed to structural characteristics of the biofilm community or to intrinsic biofilm-specific physiological programs. In order to address this longstanding question, we focused on poly-N-acetylglucosamine (PNAG)–based biofilms, a clinically relevant phenotype of many bacterial pathogens, including *E. coli*. Instead of working in a biofilm-permissive genetic background, in which the timescale of biofilm formation is slow, we applied the functionally active secreted version of the PNAG exo-polysaccharide (sPNAG) to wild-type *E. coli* cells, generating robust biofilms on the timescale of hours. In this way, we managed to uncouple upstream regulatory processes and matrix preparatory phase of biofilm formation, focusing specifically on the latter part. By using a powerful genome-wide technology, we identified the genes and pathways involved in sPNAG-based biofilm formation. Our results revealed that structural interactions between sPNAG and surface structures such as lipopolysaccharides are the crucial determinants of biofilm formation and that multiple pathways including acid-tolerance, capsule biosynthesis, and regulation of cell morphology modulate this phenotype.

## Introduction

Biofilms are an integral component in the life-cycle of many microorganisms. Compared to their planktonic complement, however, bacterial biofilms have remained poorly understood, mostly due to the inherent complexities associated with biofilm studies, including spatial heterogeneity of the biofilm structure, longer generation time, and uncharacterized growth parameters [1].

Bacterial biofilms are characterized by the presence of an extracellular polymeric matrix, which encases the cells. The physicochemical properties of this matrix, including its charge, porosity, and architecture are prominent determinants of biofilm lifestyle. The matrix, for example, could act as a protective barrier by interacting with large, charged, or reactive biocidal agents and neutralizing them [1]. One major component of matrix in various bacterial species is a homopolymer of N-acetylglucosamine. In fact, poly-*N*-acetylglucosamine (PNAG) is the major virulence factor of *Staphylococcus epidermidis* [2]. There is increasing evidence that this polysaccharide is produced by a variety of other pathogens including *Bordetella*, *Yersinia*, *Staphylococcus*, *Actinobacillai*, and certain pathogenic *Escherichia coli* strains as well. It was reported that enzymatic hydrolysis of poly-*N*-acetylglucosamines disrupts biofilm formation by *Yersinia pestis*, *Pseudomonas fluorescens*, *Aggregatibacter actinomycetemcomitans*, pathogenic *E. coli* strains, and various *Bordetella* species [3]–[7]. This suggests that PNAG is a critical component of the biofilm structure made by all these bacteria. Furthermore, a recent study showed that most *E. coli* strains isolated from urinary tract and neonatal bloodstream infections possess the *pga* locus required for PNAG biosynthesis, and almost all of them produce immunologically detectable levels of PNAG [8].

Involvement of PNAG-based biofilms in the pathogenesis of various bacterial species makes it an important phenomenon to study [4], [8]–[11]. Even though the properties of PNAG-based biofilms have been extensively studied in *Staphylococcus* species [2],[12], the existence of a PNAG-based matrix in biofilm structures from other species, including *E. coli*, has been reported only recently [3],[13], and is not as well characterized as it is in *Staphylococcus* species. However, there are some features of PNAG-based biofilm like spatial distribution of the cells in PNAG-based biofilms that are better studied in *E. coli* [14]. In *E. coli* K-12, expression of PNAG biosynthesis genes is not high enough to support the formation of a robust biofilm structure under laboratory conditions [15], complicating the analysis of this phenotype. Therefore, in order to study the genetic basis of PNAG-based biofilm formation, we decided to enhance this phenotype in *E. coli* K-12 by either increasing the level of endogenous PNAG or providing an exogenously produced form of PNAG.

PNAG production in *E. coli* can be enhanced by manipulation of the genetic elements involved in *pga* locus regulation [16],[17]. For example, *E. coli csrA* mutants overproduce the PNAG polymer [13],[17]. However, *csrA* is a master regulator of the carbon storage system, and *csrA* mutants show highly pleiotropic phenotypes. An alternative approach for enhancing PNAG-based biofilm formation would be to use a functionally active exogenous source of PNAG to induce biofilm formation in *E. coli*. In the *csrA* mutant background, cells secrete PNAG polymer into the growth media [18]. Therefore, the spent media from the *csrA* mutant culture can be used as a potential source of PNAG for inducing biofilm formation.

We observed that application of exogenously produced PNAG, isolated from *ΔcsrA* cells saturated culture of *csrA* mutants, led to robust biofilm formation in the *E. coli* K-12 background. This provided us a unique opportunity to extensively characterize the underlying genetics of the PNAG-based biofilm formation phenomenon. Given our observations, we favor a model in which electrostatic interactions between this polysaccharide and cell surface structures, such as lipopolysaccharide (LPS), are critical for PNAG-induced biofilm formation by *E. coli*. We also show that although response to PNAG polymer is a purely structural phenomenon, it can be modulated by multiple pathways including LPS biosynthesis, the acid tolerance system, capsule biosynthesis, and regulation of cell morphology.

## Results

### Biofilm-Inducing Activity of Secreted PNAG in Spent Media of *ΔcsrA* Cells

**Figure 1:**
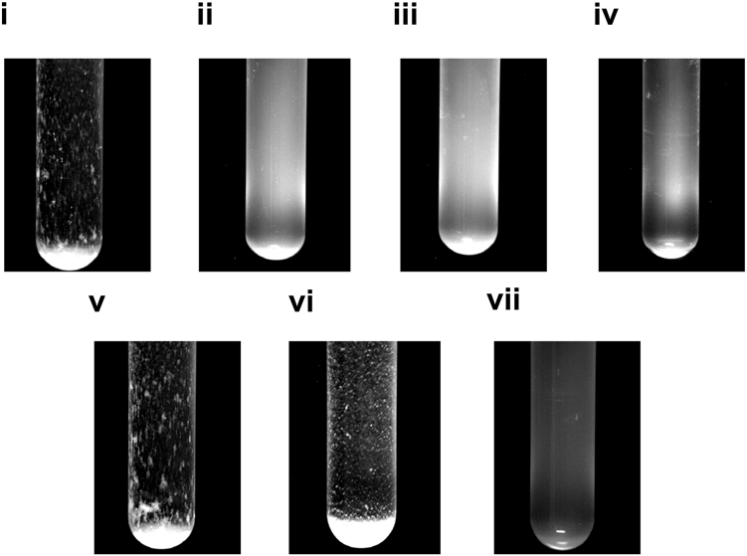
Secreted PNAG induces biofilm formation. (i) Wild-type cells in LB+sPNAG. (ii) Wild-type cells in LB. (iii) *ΔrfaY* cells in LB+sPNAG. (iv) Wild-type cells in PBS. (v) Wild-type cells in PBS+.2%Glucose+sPNAG. (vi) Wild-type cells in PBS+.2% Lactose+sPNAG. (vii) *ΔlacZ* cells in PBS+sPNAG+lactose. In the experiments carried out in the presence of lactose, since there was no pre-induction phase with lactose, the observed cell density was lower.

*Escherichia coli csrA* mutants secrete poly N-acetylglucosamine polysaccharide into the culture medium [18]. In order to see whether the secreted polysaccharide is functional, we grew *ΔcsrA* cells to stationary phase, discarded the cells, and added the cell-free spent media to wild-type MG1655 cells in the presence of fresh media. Interestingly, we observed that the cell-free spent media of saturated *ΔcsrA* cultures made wild-type cells form a biofilm on a rapid time-scale (Figure 1, compare i and ii). Presence of a carbon source, as expected, was required for formation of mature visible microcolonies by living wild-type cells (Figure 1, compare iv and vii to v and vi). Synthesis of PNAG in *E. coli* requires the gene products of the *pgaABCD* operon [13],[17]. To test whether the observed biofilm-inducing activity is associated with the secreted PNAG (sPNAG), we generated four double mutants each harboring *csrA* deletion together with the deletion of one of the four genes present in the *pga* locus. There was no detectable biofilm-inducing activity in the cultures of any of the four double mutants. Furthermore, the biofilm-inducing activity was lost after treatment of *ΔcsrA* culture spent media with Dispersin B, an enzyme that specifically cleaves PNAG [3],[5],[19], confirming that the biofilm inducing factor is an N-acetylglucosamine containing polysaccharide. Differential up-regulation of the *pga* locus transcription between the *ΔcsrA* and wild-type cells was also confirmed by a reporter assay (Figure S1).

**Figure 2:**
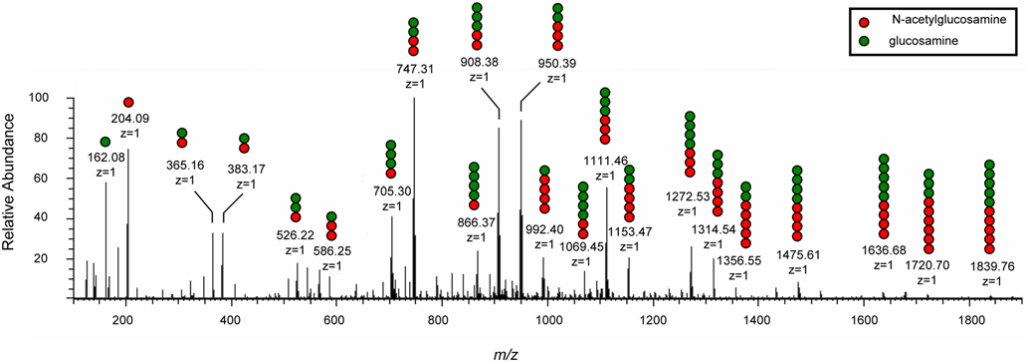
ESI LTQ OrbiTrap mass spectrum of digested sPNAG, acquired in positive mode. Purified sPNAG was digested with Dispersin B in water and analyzed by mass spectrometry after dialysis. For simplicity, only the most prominent peaks are labeled with their *m/z* and *z* values (where *z* is the charge). For each labeled peak, molecular composition was schematically illustrated by red and green circles representing acetylated and non-acetylated saccharide units, respectively (e.g., two red and three green circles corresponds to a pentamer with two N-acetylglucosamine and three glucosamine residues). For all identified molecules, the ion-pairs corresponding to the singly-charged protonated ions and their dehydration products were detected (e.g., 383.17 and 365.16). As shown in the figure, all prominent peaks correspond to a partially de-acetylated N-acetylglucosamine oligomer or the monomers. Almost all unlabeled peaks correspond to one member of some dehydrated-nondehydrated ion pairs, doubly charged versions of some of the expected molecules, or some adducts. A complete list of *m/z* values for all potential mono- and oligosaccharides species that could be generated from an incomplete digestion of a PNAG sample with all possible acetylation patterns is also given in Table S1, as a reference.

In order to confirm that the observed biofilm formation phenotype is specific to sPNAG and rule out the possibility of involvement of other biofilm-inducing agents that might be present in the *ΔcsrA* spent media, we purified sPNAG from spent media. As part of the purification steps, sPNAG was treated with various enzymes, including DNase, RNase, α-amylase, and Proteinase (see Materials and Methods section). The purified sample showed identical biofilm-inducing activity, suggesting that sPNAG is sufficient for inducing biofilm formation. The purified polysaccharide was also characterized by mass spectrometry. As shown in Figure 2, almost all prominent molecules found on the mass spectrum corresponded to N-acetylglucosamine oligomers with different levels of acetylation or to their monomers. In *S. epidermidis*, deacetylation of PNAG polymer introduces positive charges in the otherwise neutral polymer [20]. Our results also indicate that a considerable fraction of sPNAG building units is deacetylated, which should leave a net positive charge on the polymer. Purified PNAG isolated from *Staphylococcus aureus* strain MN8m [21] showed similar biofilm-inducing activity when applied to wild-type MG1655 cells, further confirming that PNAG is sufficient for the observed biofilm-inducing activity. The presence of various identical peaks in the mass spectra of PNAG from *E. coli* and *S. aureus* (Figure S2) indicates that they are closely related molecular species.

The response of wild-type *E. coli* cells to sPNAG was so fast that we decided to study the early stages of the process. Using time-lapse microscopy (Video S1), we observed that wild-type cells started seeding microcolony structures on a glass slide in less than an hour of exposure to sPNAG. The pace of microcolony formation observed here was much faster than the previously reported behavior by *csrA* mutants [15]. The microcolonies expanded in size due to both growth of pre-existing cells and continued incorporation of new cells. No similar activity was observed in the absence of sPNAG (Video S2). These results show that sPNAG enhances both cell-cell and cell-surface interactions. SEM images of the biofilm structures formed by wild-type cells in the presence of sPNAG also confirmed the presence of an extracellular matrix encasing the cells (Figure S3).

### Genome-Wide Identification of Loci Involved in sPNAG-Based Biofilm Formation

**Figure 3:**
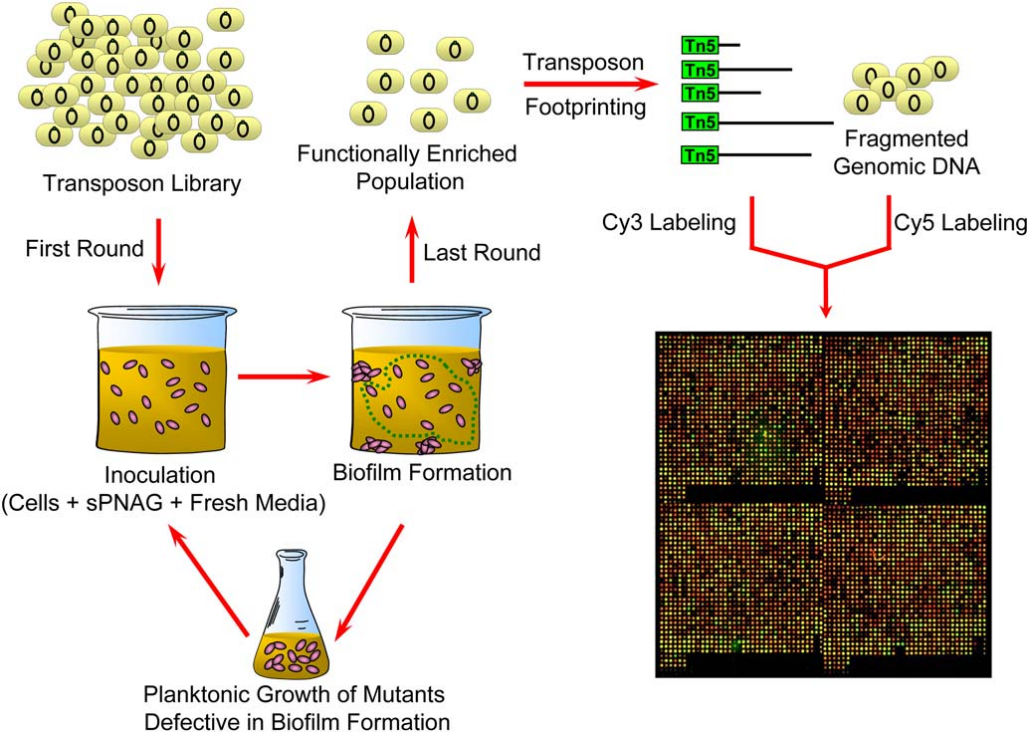
Schematic representation of the enrichment procedure for transposon mutants defective in sPNAG-based biofilm formation.

We used a microarray-based genetic footprinting strategy [22] to study the genetic basis of sPNAG-mediated biofilm formation. Since formation of biofilm by wild-type cells in the presence of sPNAG is robust and fast, the phenotype is highly amenable to genetic analysis. Starting from a close to saturation Tn5-based library of approximately 5×10^5^ independent transposon insertional mutants, generated in wild-type *E. coli* MG1655, we devised a selection strategy to enrich for mutants defective in responding to sPNAG. Roughly 10^10^ cells of the abovementioned library were exposed to sPNAG in LB medium. After 12 hours of incubation, cells which were present in the liquid phase of the culture and were not part of the biofilm were isolated, grown up to log phase, and transferred to a new container with fresh media and fresh sPNAG, in order to enrich for mutants impaired in responding to sPNAG. After four rounds of serial enrichment, no visible biofilm formation activity was present in the enriched population. A schematic representation of the enrichment procedure is shown in Figure 3.

**Figure 4:**
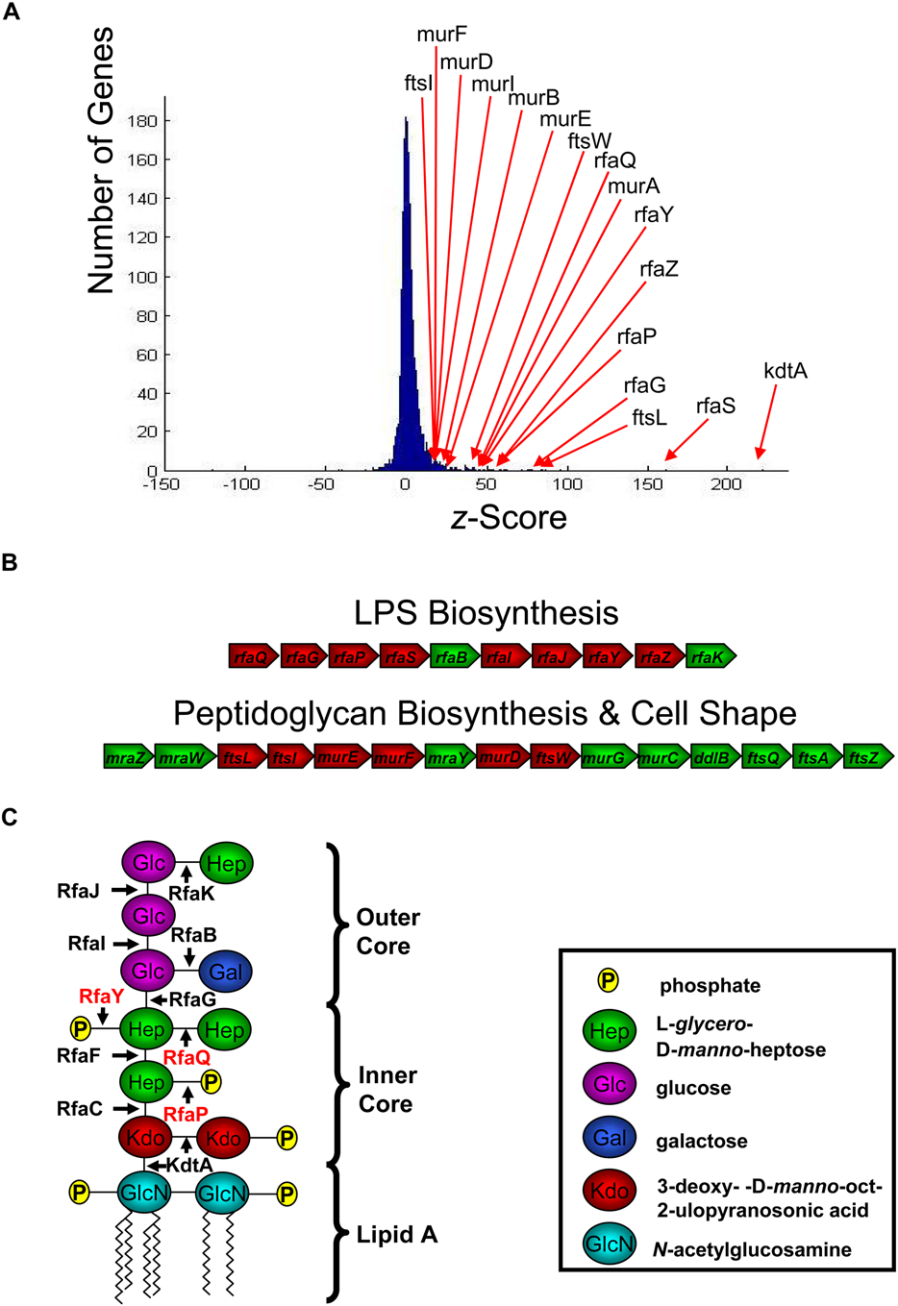
Genome-wide identification of loci involved in sPNAG-based biofilm formation. (A) Distribution of *z*-scores after enrichment for mutants impaired in sPNAG-based biofilm formation. A transposon insertion mutant library of *E. coli* has been enriched for mutants that are defective in responding to sPNAG. To quantify the contribution of different loci to this phenotype, the insertion sites of the transposon in the enriched population were mapped using a microarray-based approach. The average signal acquired for each ORF on the microarray from two replicate experiments was used to calculate the *z*-score value for that ORF. This value reflects the relative abundance of transposon insertion events in the ORF (or in its vicinity) in the enriched population compared to the maximally diverse parental library. Distribution of these *z*-scores in the enriched population is illustrated by a histogram. Some of the ORFs which were significantly enriched were labeled on the histogram. More detailed information regarding the calculation of *z*-score is provided in Dataset S1. (B) Many of the transposon insertions with high *z*-scores in the first selection belonged to one of the two long gene clusters involved in LPS biosynthesis (top row) or regulation of cell shape and peptidoglycan biosynthesis (bottom row). ORFs with high *z*-scores in the selection are shown in red. (C) Structure of the major glycoform of *E. coli* K-12 LPS [26] together with its biosynthetic genes (enzymes). The three genes (enzymes) whose deletion abolished sPNAG-based biofilm formation are highlighted in red.

To quantify the contribution of different loci to this impaired biofilm formation phenotype, the insertion sites of the transposon in the enriched population were mapped using a microarray-based approach [22]. The histogram in Figure 4A shows the normalized average output of the hybridization data from two experimental replicates. The *z*-score for each ORF is indicative of the abundance of transposon insertion events in that ORF (or its vicinity) in the enriched population of mutants. More detailed information regarding the calculation of *z*-score is provided in the supporting information section (Dataset S1). Interestingly, the majority of the genes that were highly enriched in our selection were involved in two major biological processes: LPS core biosynthesis and regulation of cell shape and morphology. Most of these candidate genes belong to two long operons (Figure 4B). In other words, genetic perturbations caused by transposon insertion in many components of LPS biosynthesis or cell shape regulation made wild-type cells lose their ability to form a biofilm in the presence of sPNAG. The dominance of genes involved in the synthesis and regulation of exposed structural components suggested that physical interaction between sPNAG and these surface structures may be a major determinant of biofilm formation capacity. sPNAG pre-treatment of the cells, however, did not cause any change in the migration of their extracted LPS samples on SDS-PAGE gels (Figure S4).

Incomplete disruption of the targeted genes and polar effects are characteristics of transposon insertion events. In order to get around these complications and get a fine-scale perspective of genetic perturbations that prevent cells from responding to sPNAG, we generated in-frame deletions of some of the candidate ORFs, obtained from our transposon mutagenesis screen, in the MG1655 background [23],[24]. We then studied the behavior of these mutant strains in the presence of sPNAG (Table S2). Among the candidate genes, deletion of *rfaY*, *rafP*, or *rfaQ*, diminished sPNAG-based biofilm formation (Figure 1, compare i and iii). As shown in Figure 4C, the product of these three genes are directly or indirectly involved in addition of phosphate groups to the inner core of LPS [25].

Since the major common point in the LPS structure of *rfaY*, *rfaP*, and *rfaQ* mutants is the lower density of negative charge (phosphate groups) on their LPS outer core, these phosphate groups are likely to be critical for this interaction. Given the positive charge of sPNAG, we favor a model in which electrostatic interaction between the positively charged polysaccharide and the negatively charged phosphate groups on LPS is the major determinant of sPNAG-mediated biofilm formation. Electrostatic interactions were also proposed to be responsible for PNAG-based biofilm formation in *S. epidermidis* [20].

It is postulated that neighboring LPS molecules can be cross-linked by divalent cations due to the presence of phosphate groups in the LPS structure [25]. Therefore, any presumable electrostatic interaction between phosphate groups and sPNAG should be sensitive to increasing concentrations of divalent cations. As shown in Table S3, response to sPNAG is lost in Ca^2+^ concentrations higher than 100 µM, which could be considered as an additional support for our electrostatic interaction model. However, changing calcium concentration might also change cell viability. Exposure to this concentration of calcium, however, did not have any effect on the viability of the cells, as measured by viable counting and CFU determination. Identical results were obtained when the experiment was repeated with other divalent cations (manganese or magnesium).

Based on the microarray, transposon insertions in genes involved in regulation of cell shape and morphology should also interfere with sPNAG-base biofilm formation. However, since all the genes in this category are essential, we could not introduce those deletions into the wild-type background and check their phenotype. Transposon insertion events in this operon presumably occurred in either regulatory regions (e.g. promoter) or dispensable parts of essential genes.

As could be inferred from the data, there was no significant correlation between capacity of producing PNAG and responding to it. However, in order to confirm this, we studied sPNAG-induced biofilm formation phenotype of all four *ΔcsrA Δpga* double-mutants (*ΔcsrA pgaA::kan*, *ΔcsrA pgaB::kan*, *ΔcsrA pgaC::kan*, and *ΔcsrA pgaD::kan*) and also all four *Δpga* single-mutants and found it to be indistinguishable from that of wild-type cells.

### Systematic Characterization of Extra-Genic Suppressors Reverting the Biofilm Formation Defect of *ΔrfaY* Cells

We were curious to know whether deletion of *rfaY*, *rfaQ*, or *rfaP* abolished the response to sPNAG due to downstream signaling events or the phenotype was a simple consequence of the structural modifications imposed on LPS. Therefore, we decided to systematically identify extra-genic suppressors which can restore biofilm formation capacity of *ΔrfaY* cells and test whether there are any known or putative signaling pathway components among such suppressors.

**Figure 5:**
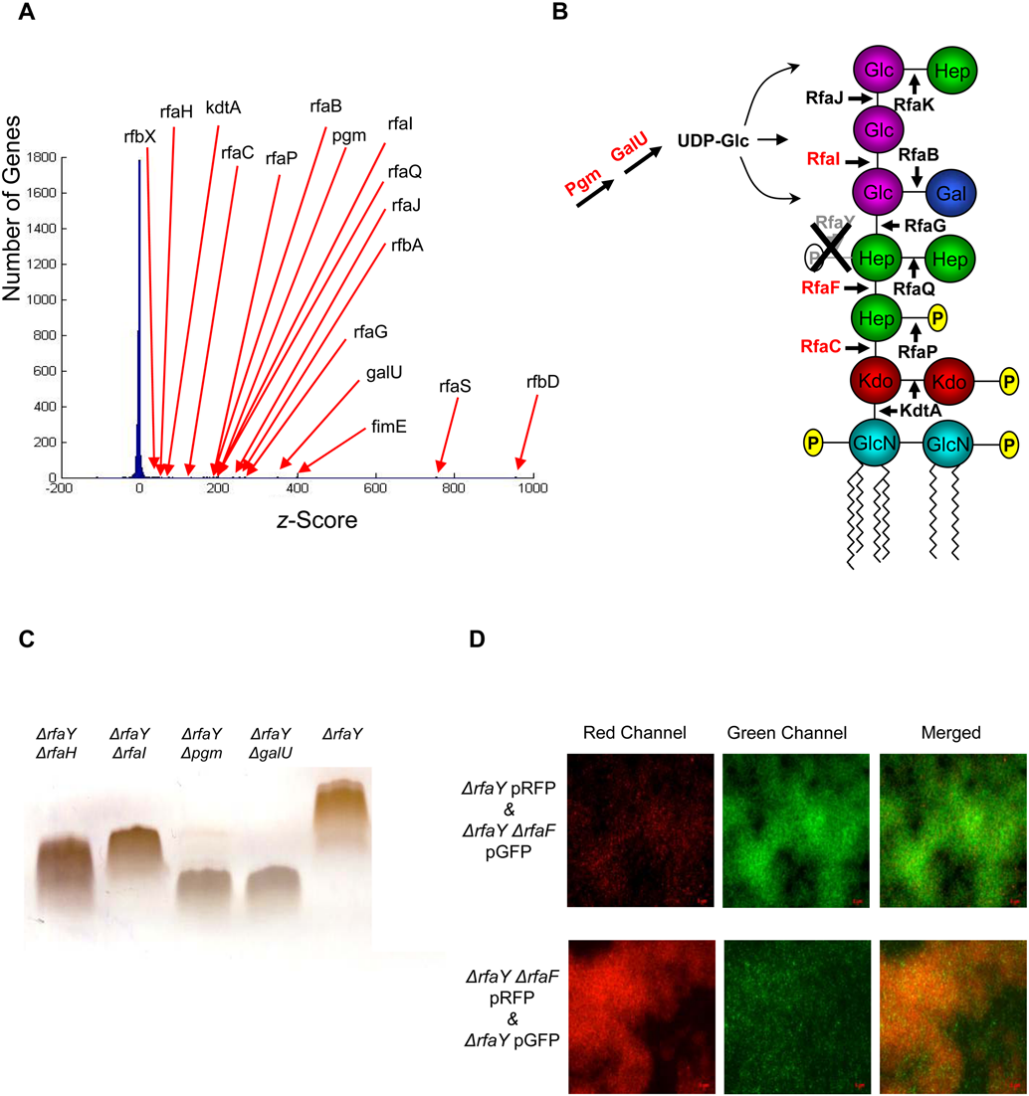
Characterization of extra-genic suppressors reverting the biofilm formation defect of *ΔrfaY* cells in a transposon mutagenized library. (A) Distribution of *z*-scores after enrichment for *ΔrfaY* double mutants that recovered the capacity for sPNAG-based biofilm formation. (B) Schematic representation of LPS structure, together with its biosynthesis genes (enzymes) in *ΔrfaY* cells. Secondary mutations which reverted the biofilm-formation deficiency of the *ΔrfaY* cells are highlighted in red. (C) LPS samples from a subset of *ΔrfaY* double mutants that recovered their ability to respond to sPNAG are separated on a SDS-PAGE gel. All double mutants showed truncated versions of LPS compared to the *ΔrfaY* cells. LPS samples from *ΔrfaY ΔrfaC* and *ΔrfaY ΔrfaF* cells were not detectible on the gel. (D) *ΔrfaY* cells, expressing RFP (mCherry), and *ΔrfaY ΔrfaF* cells, expressing GFP, were competed against each other for biofilm formation on a glass slide surface in presence of sPNAG (top row). Three images from left to right show the red channel, green channel, and the merged version. Similar results were obtained after swapping the fluorescent markers (bottom row).

To identify suppressors of *rfaY* deletion, a transposon insertion library was generated in the *ΔrfaY* background (i.e. a strain with clean deletion of *rfaY* ORF), and enriched for double mutants that recovered their ability to form a biofilm in the presence of sPNAG (opposite to what was demonstrated in Figure 3). A glass slide was provided as the biofilm formation surface and at the end of each round, the slide was transferred to a new container with sPNAG and fresh media. After four rounds of enrichment, macroscopic microcolony structures could be detected on the glass slide. The transposon insertion sites in the enriched population were mapped by the same footprinting strategy described previously (Dataset S2). Surprisingly, transposon insertions in many of the LPS biosynthetic genes were significantly enriched in this selection (Figure 5A)

To validate our microarray predictions, we generated in-frame deletions of the candidate genes in the *ΔrfaY* background and studied their behavior in the presence of sPNAG (Table S4). We found that deletion of *rfaC*, *rfaF*, *rfaI*, *pgm*, *galU*, *rfaH*, *rfbD*, *rfbC*, and *adiY* reverted the phenotype of *ΔrfaY* cells. *rfaC*, *rfaF*, *rfaI*, *pgm*, *rfbC*, *rfbD*, and *galU* are involved in the synthesis of LPS core structure or its precursors (Figure 5B). *rfbC* and *rfbD* are involved in rhamnose biosynthesis which is a component of the second major LPS glycoform in *E. coli* K12 [26], which is not shown in Figure 5B. *rfaH* is a transcription anti-terminator which is required for full-length transcription of long operons, including *rfaQ-K* operon [27]. *adiY* is the positive regulator of the arginine decarboxylase system and will be discussed later. Overall, these mutants make truncated versions of LPS, an expectation we verified for a subset of them (Figure 5C). We hypothesized that in these truncated structures, inner phosphate groups of lipid A or possibly other negatively charged cell-surface moieties (which tend to be buried by longer LPS chains in the wild-type cells) are now more exposed and available for interaction with sPNAG. Deleting any of the four genes in the *pga* operon did not restore the biofilm formation capacity of the *ΔrfaY* cells, as was also inferred from the microarray data.

We also used fluorescence microscopy to characterize the dynamics of biofilm formation in a heterogeneous population composed of cells either capable (represented by *ΔrfaY ΔrfaF* cells) or defective (represented by *ΔrfaY* cells) in biofilm formation. To this end, *ΔrfaY* cells, expressing RFP fluorescent marker, were competed against *ΔrfaY ΔrfaF* cells expressing GFP with the starting ratio of 1∶1 for making a biofilm on a glass slide in the presence of sPNAG. After 12 hours, the biofilm structure formed on the glass slide was visualized by fluorescence microscopy. As shown in Figure 5D, top row, microcolonies in biofilm structure were mostly formed by *ΔrfaY ΔrfaF* double mutants (i.e. GFP expressing cells). The same result was obtained by swapping the fluorescent labels (Figure 5D, bottom row).

**Figure 6:**
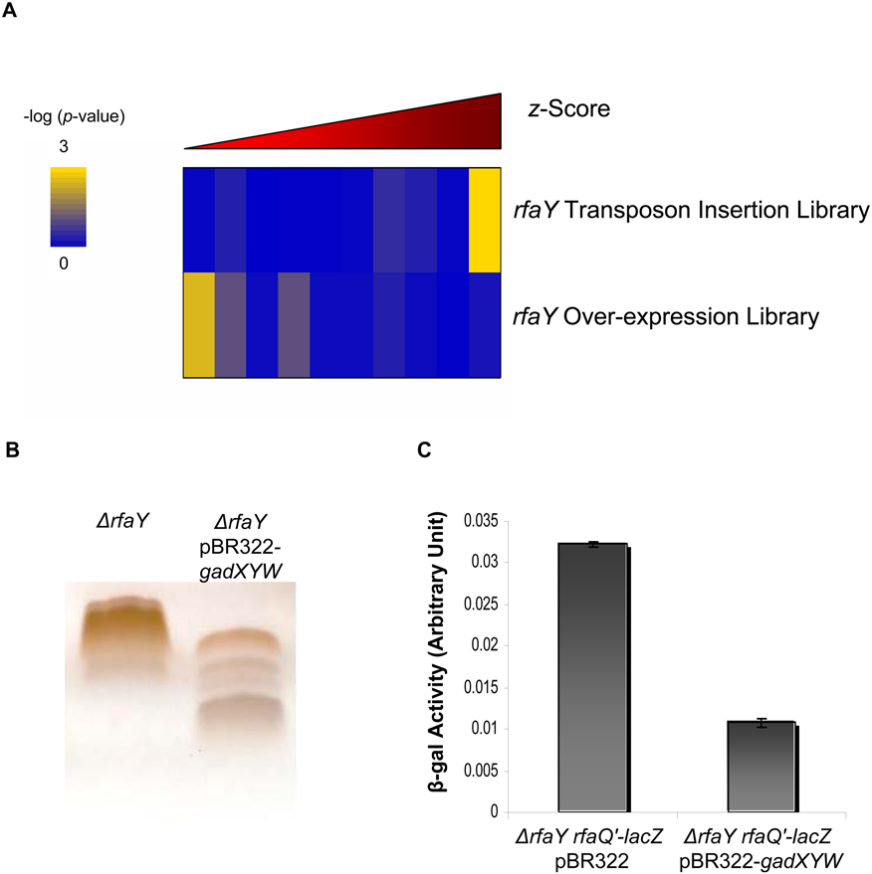
Characterization of extra-genic suppressors reverting the biofilm formation defect of *ΔrfaY* cells in an over-expression library. (A) Distribution of LPS biosynthesis genes were compared in the *ΔrfaY* transposon insertion and over-expression libraries. The microarray outputs of the *ΔrfaY* transposon insertion and the *ΔrfaY* over-expression libraries were sorted separately based on their *z*-score and divided into 10 equally populated bins. The number of genes belonging to lipopolysaccharide biosynthesis gene cluster (GO index GO:0009103) in each bin was counted and used to calculate hypergeometric *p*-value for over-representation of LPS biosynthesis genes in that bin for each library. Each bin was color-coded based on its −log_10_(*p*-value). The yellow color for a bin reflects statistically significant abundance of lipopolysaccharide biosynthesis genes in that bin. As shown, many LPS biosynthetic genes were found to be among the top 10% highly enriched category in the *ΔrfaY* background transposon insertion library, while in the over-expression library they belonged to the top 10% most depleted group. (B) LPS samples extracted from *ΔrfaY* pBR322-*gadXYW* and *ΔrfaY* cells and separated on SDS gels. (C) Transcription level of the *rfaQ-K* operon promoter is compared between *ΔrfaY* pBR322-*gadWYX* and *ΔrfaY* pBR322 cells by a β-galactosidase assay.

Transposon insertions typically lead to a loss of function phenotype. In order to complement our transposon insertion based approach, we used an over-expression library in the *ΔrfaY* background. This library contained ∼2.5×10^5^ independent mutants each carrying a 1–3 kb long genomic fragment of *E. coli* cloned into the pBR322 plasmid. The over-expression library was enriched for mutants responding to sPNAG, similar to the approach used for studying the *ΔrfaY* transposon insertion library. After four rounds of enrichment, the over-expressed fragments represented in the enriched population were identified by microarray hybridization (Dataset S3). As expected, when comparing the results from *ΔrfaY* transposon insertion library with *ΔrfaY* over-expression library, LPS biosynthesis genes showed the opposite behavior (Figure 6A). Many LPS biosynthetic genes like *rfaG*, *rfaJ*, *rfaI*, *rfaP*, *kdtA*, *rfbX*, *rfaZ*, and *rfaL* were found to be among the top 10% highly enriched category in the *ΔrfaY* background transposon insertion library while in the over-expression library, they belonged to the top 10% most depleted group.

The microarray results also revealed that mutants over-expressing certain genes associated with the acid tolerance system in *E. coli* were abundant in the enriched population. In order to clarify how this system contributes to biofilm formation, we further characterized the phenotypic consequences of over-expressing these genes in individual cells. To this end, we isolated individual clones from the enriched population. One of the isolated mutants was found to have the genomic region corresponding to three genes, *gadW*, *gadY*, and *gadX*. GadX and GadW are dual regulators of the glutamate-dependent decarboxylase acid-resistance system of *E. coli*. [28]. GadY is a small RNA which acts as a positive regulator of *gadX* [29].

Our previous results demonstrated that LPS modification was a dominant mechanism in regulating response to sPNAG. Therefore, we first investigated the effect of *gadXYW* over-expression on LPS structure. As shown in Figure 6B, *ΔrfaY* pBR322-*gadWYX* strain contained some smaller LPS variants as compared to the parental *ΔrfaY* cell. This suggests that the reversion of *ΔrfaY* phenotype upon over-expression of *gadWYX* gene cluster is a consequence of this truncated LPS structure. To test whether this change in LPS structure was due to a transcriptional regulatory event, we measured the transcription of the *rfaQ-K* operon in both pBR322-*gadWYX* and pBR322 (empty vector) backgrounds by a β-galactosidase assay and found it to be almost 3-fold lower in the *gad*-over-expressing cells (Figure 6C). Evidence regarding the existence of a cross-talk between acid tolerance system and LPS regulation has been observed before, and *gadE*, the transcriptional regulator of the acid resistance system in *E. coli*, was reported to be a potential activator of the *rfaQ-K* operon [30]. We also found that over-expression of *gadY* alone was sufficient to cause the phenotype, although not as strongly as the over-expression of *gadXYW*.

A strain harboring the pBR322-*argR* plasmid was also isolated from the enriched *ΔrfaY* library. However ArgR, the negative regulator of arginine biosynthesis system, acts as a weak suppressor of *ΔrfaY* biofilm formation deficiency. Putting all these observations together, four of the suppressors found in the transposon insertion and over-expression libraries, *adiY*, *gadX*, *gadW*, and *argR*, were directly or indirectly associated with the amino acid decarboxylase systems, involved in acid tolerance in *E. coli*. The biological function of these suppressors (Table S5) and the distribution of acid tolerance genes in the over-expression library (Figure S5) suggest that down-regulation of the acid stress response, or more specifically the amino acid decarboxylase systems, positively contribute to biofilm formation in the *ΔrfaY* background, presumably due to the changes imposed on LPS.

### A Model for sPNAG-Mediated Biofilm Formation: From Passive Nucleation to Active Maturation

Overall, our observations support the existence of a physical interaction between sPNAG and LPS. As such, biofilm formation in the presence of sPNAG may be a purely structural phenomenon, occurring as a simple consequence of passive interactions between sPNAG and LPS. Based on this model, even dead cells with intact outer membrane structure should still be capable of responding to sPNAG. To test this, we killed wild-type cells by either UV-irradiation or exposing them to formaldehyde, and visualized their behavior upon exposure to sPNAG. Time-lapse microscopy showed that these dead cells start nucleating microcolony structures, similar to living cells (Video S3). All together, our observations argue that sPNAG-mediated biofilm formation can be considered as a two-step process, starting with the nucleation event which is a purely structural phenomenon, followed by microcolony expansion and maturation which is a growth-dependent process (Figure 1, compare iv and vii to v and vi).

### From Laboratory Strains to Clinical Isolates

Most natural and clinical isolates of *E. coli* produce different serotype-specific surface structures including O-antigen and capsular polysaccharide, also known as K-antigen, which are absent in *E. coli* MG1655 [31],[32]. We were curious to know how variations in composition of these surface antigens might affect the response to sPNAG. Therefore, we chose 11 strains, which were reported to be competent of endogenous PNAG production [8], for further analysis. Among these strains, 7 formed biofilms in the presence of sPNAG (Table S6), and two of the latter group were also O-antigen^−^ (Figure S6). In case of K-antigen, we focused on K1 capsule, a homopolymer of α-(2-8)-linked polysialic acid [33], which is the predominant capsule found in a major subset of these clinical isolates. Three K1^+^ isolates used in this study were also capable of responding to sPNAG (Table S6).

Natural and clinical isolates of *E. coli* possess uncharacterized surface structures other than K1, e.g. fimbriae and other capsular polysaccharides. These structures could significantly affect the physicochemical properties of the cell surface. Consequently, cell response to sPNAG in these clinical isolates could not be solely judged based on their O-antigen structure or presence of K1 capsule. Furthermore, the limited number of strains tested in this study and their non-isogenic background make these observations preliminary and they should be followed up with future studies. Therefore, we decided to study the role of O- and K-antigen in the well-characterized K-12 background.

We generated all possible combinations for presence or absence of O16 antigen and K1/K92 capsule in the K-12 background. K92 is a polysialic acid capsule very similar to K1, and its biosynthetic gene cluster can be transferred on a plasmid. As shown in Table 1, only non-capsulated O16^+^ cells were impaired in sPNAG-based biofilm formation. In the presence of both O16 and K1/K92 antigen, however, cells were capable of responding to sPNAG, which is not surprising considering that capsule is a more exposed surface structure than O-antigen [32]. Since K1 and K92 capsules confer a high density of negative charge to the *E. coli* cell surface, their presence could contribute to establishing any potential electrostatic interaction with sPNAG. These data suggest that loss of O-antigen (O16) or presence of K1 capsule is associated with sPNAG-induced biofilm formation in the *E. coli* K-12. However, in order to confirm the involvement of these structures in sPNAG-induced biofilm formation, targeted genetic experiments together with more careful characterization of their role in physiochemical properties of outer membrane are required.

**Table 1:** Role of surface antigens in sPNAG-based biofilm formation.

	Presence (+) or Absence (−) of O (O16) or K (K1/K92) Antigen					
**O-Antigen**	−	+(O16)	−	−	+(O16)	+(O16)
**K-Antigen**	−	−	+(K1)	+(K92)	+(K1)	+(K92)
**Response to sPNAG**	+	−	+	+	+	+

O-antigen production was restored in MG1655 by plasmid pMF19, encoding a functional copy of rhamnosyl-transferase, RfaL, whose gene is mutated in *E. coli* K-12 [31]. Transformation of MG1655 strain with this plasmid allowed production of O16 antigen [45] confirmed on SDS-PAGE gels (Figure S6, compare lane 8 and 9). For K-antigen, only K1 and K92 capsules were considered. In the case of the K92 capsule, the gene cluster responsible for its biosynthesis was cloned into the pGB20 plasmid and could be easily transferred to any background [46]. As a K1^+^ cell, a K-12-based K-12/K1 hybrid (EV36), generated by conjugation, was used which was capable of producing a polysialic acid capsule indistinguishable from that of natural K1 strains [47]. All the experiments were performed in the MG1655 background except for those corresponding to O16^−^K1^+^ and O16^+^K1^+^ strains, which were in the EV36 background.

### sPNAG-Based Biofilms: What Do Biofilms Share in Common?

**Figure 7:**
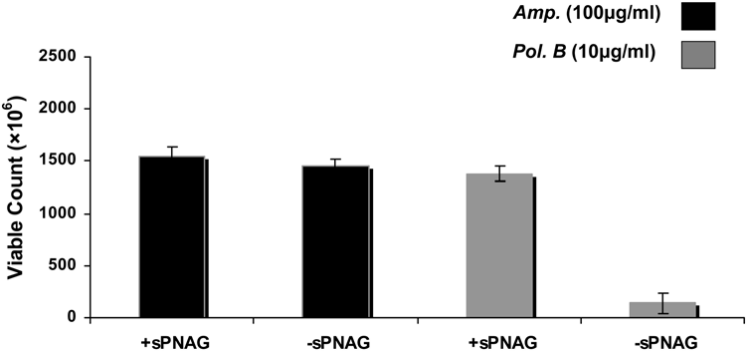
Antibiotic tolerance in sPNAG-based biofilms. 10^8^ stationary phase wild-type cells were used to inoculate fresh LB in the presence (first and third column from left) or absence (second and fourth column) of 0.1 U/ml of sPNAG. After 12 hours, polymyxin B or ampicillin was added to a final concentration of 10 µg/ml and 100 µg/ml, respectively. After 18 hours, cells were pelleted down, washed with PBS three times, treated with Dispersin B to break the biofilm structures (if any), and plated for CFU determination. Independent experiments showed that Dispersin B treatment did not change the viability of the cells. For each column, results from ten independent samples were averaged and reported. Error bars indicate the standard error of the mean calculated from all independent measurements.

Biofilms can afford protection from a variety of environmental challenges including phagocytosis, extreme pH, and antibiotic exposure [1]. We were curious to characterize some of these biofilm-specific features in sPNAG-based biofilms. In order to investigate their antibiotic tolerance, we challenged the cells with two different antibiotics: ampicillin and polymyxin B. As shown in Figure 7, cells in the context of sPNAG biofilm showed ∼10 fold higher tolerance to polymyxin B compared to planktonic cells, whereas no significant difference was observed in tolerance to ampicillin. Polymyxin B resembles antimicrobial peptides, an integral component of the innate defense system in many organisms, in terms of both structure and mechanism of action [34]. Considering sPNAG as a positively charged matrix encasing the cells, electrostatic repulsion between this polysaccharide and polymyxin B could potentially protect the bacteria by reducing the local concentration of the drug in the vicinity of the cells. However, we still consider the involvement of other as yet unknown sPNAG-induced physiological response in this phenomenon.

Considering other biofilm-specific phenotypes, we found no significant change in the rate of F-plasmid conjugation in sPNAG-based biofilms (Figure S7). We also did not observe any evidence supporting the existence of a phase-variable mechanism regulating sPNAG production in *E. coli* (Figure S8 and Figure S9).

We were also curious whether sPNAG can induce biofilm formation in species other than *E. coli*, because this could potentially facilitate interspecies interactions, e.g. conjugation, between *E. coli* and other microorganisms. Therefore, we exposed *Salmonella typhimurium* LT2 cells to sPNAG similar to what was done for *E. coli*, but we did not observe any detectable biofilm formation. We reasoned that if sPNAG-mediated biofilm formation has a structural basis, then a mutant *Salmonella* with modified surface characteristics may be capable of forming a biofilm in the presence of sPNAG. To test this hypothesis, we applied sPNAG to a transposon insertion library of *S. typhimurium* LT2 with approximately 10^5^ mutants and enriched for mutants that form biofilms. After four rounds of enrichment, macroscopic microcolony structures were formed by cells. We mapped the transposon insertion sites in 4 of the mutants capable of forming biofilms in a sPNAG-dependent pattern. In all cases, the transposon mapped to different positions in the *rfaK* gene, involved in LPS biosynthesis. *Salmonella rfaK* mutants are lacking most of the O-antigen structure [35], providing further support, beyond *E. coli*, for our physical interaction model between sPNAG and LPS.

## Discussion

Poly-*N*-acetylglucosamine, the major virulence factor of *Staphylococcus epidermidis*, has recently been found in many other pathogenic bacteria [7], including *E. coli*, but PNAG-based biofilms in these pathogens are poorly characterized relative to *Staphylococcus* species [2]. Here, we carried out a systematic genetic analysis of poly-*N*-acetylglucosamine-induced biofilm formation in *E. coli*. However, instead of working in a biofilm-permissive genetic background, in which the time scale of biofilm formation is slow, we applied the functionally active secreted version of PNAG (sPNAG) to wild-type *E. coli* MG1655 cells and observed rapid and reproducible biofilm formation. The fast kinetics and robustness of sPNAG-induced biofilm formation phenomenon allowed us to comprehensively characterize its underlying genetic basis. Our observations support the notion that electrostatic interaction between positively charged sPNAG and different cell surface antigens with negative charge is responsible for the formation of the biofilm structure. This is consistent with the generally accepted intuition that physicochemical properties of the matrix, including its charge, geometry, ion selectivity, and pore size contribute significantly to biofilm formation [1],[18].

The composition and physicochemical properties of cell surface structures can be modulated by multiple biological pathways and environmental factors. Therefore, although response to sPNAG seems to be the consequence of simple electrostatic interactions, it can be regulated by a complex interplay between LPS structural dynamics, the presence of serotype-specific capsular polysaccharide, the acid tolerance system, and cell morphology as shown in this work. There are also other relevant pathways, not fully active in our laboratory strain, that might contribute to this phenotype, such as addition of positively charged 4-amino-4-deoxy-L-arabinose to lipid A involved in polymyxin B and other cationic antimicrobial peptide tolerance in *E. coli* and *S. typhimurium*. Interestingly, a polymyxin B resistant mutant isolated from our transposon insertion library was also impaired in responding to sPNAG (data not shown). Since resistance to polymyxin is usually concomitant with higher density of positive charge on the outer membrane [36], polymyxin resistant mutants are expected to be defective in interacting with sPNAG. Given that antimicrobial peptides generated by the host immune system are a major challenge for the survival of pathogens, encasement in a positively charged matrix or biofilm serves as a protective mechanism against antimicrobial peptides for sensitive cells, while resistant cells can survive without it.

Since in this study, selections were performed with pools of mutants rather than with clonal populations, phenotype of different mutants should be interpreted as a spectrum of different capacities for forming a biofilm rather than a simple binary phenomenon. The results of our selection, carried out on the over-expression library in the *ΔrfaY* background, strongly support this notion. *ΔrfaY* cells are defective in responding to sPNAG, so it is reasonable to assume that only a handful of genes in the over-expression library should be capable of suppressing its phenotype and the majority of the library mutants should be equally defective in biofilm formation. However, the fact that a considerable fraction of genes involved in LPS biosynthesis were significantly depleted in that selection (Figure 6A) indicates that even *ΔrfaY* cells might have residual biofilm formation activity and that over-expression of some LPS biosynthetic genes might reduce this partial activity. The weak biofilm formation activity of *ΔrfaY* cells could be due to their entrapment in the microcolony structures formed by other mutants in the population. This is in part shown in Figure 5D in which *ΔrfaY* cells are mostly colocalized with microcolonies rather than being uniformly distributed. Imposed modifications in LPS structures caused by over-expression of some LPS biosynthetic genes [37] might interfere with this partial activity.

In this study, we have demonstrated the involvement of different surface structures and regulatory systems in sPNAG-mediated biofilm formation. However, there might exist a myriad of other uncharacterized regulatory systems, surface structures, and environmental factors that may influence this phenotype through changes in the physicochemical properties of the cell surface. Covalent modifications of lipid A with phosphoethanolamine in a Ca^2+^-dependent manner, or with 4-amino-4-deoxy-L-arabinose in response to Mg^2+^ and pH, represent just a few examples [38]. Therefore, even if a natural isolate of *E. coli* is incapable of responding to sPNAG in the laboratory environment, it may show a different phenotype in its natural environment. There is also the possibility of higher level cooperation between different cell types in the population, with one subpopulation producing sPNAG while another is responding to it. The producer subpopulation may even be defective in the initiation of the biofilm formation process, but could be incorporated into preformed structures. Examples of population heterogeneity in biofilm communities has been reported before [1]. Furthermore, it is likely that different cells produce different versions of the PNAG polymer, with different degrees of acetylation [39] or different polymer length distributions, which could make response to sPNAG more strain-specific. A better understanding of sPNAG-based biofilms acquired from studies like this could be useful for development of new therapeutic strategies against pathogens that use this polysaccharide as a virulence factor. The systematic framework presented in this study, along with the acquired insights, should also benefit the study of microbial biofilms formed by other species.

## Materials and Methods

### Strains, Media, and Microbiological Techniques

All strains used in this study are listed in Table S7, bacteriophages and plasmids are mentioned in Table S8. All the experiments were performed in LB (1% tryptone, 0.5% yeast extract, 1% NaCl), supplemented as required with the following antibiotics: ampicillin, 50 µg/ml; tetracycline, 25 µg/ml; spectinomycin, 100 µg/ml; chloramphenicol, 30 µg/ml and kanamycin, 100 µg/ml, unless otherwise mentioned. β-galactosidase measurements were performed in triplicate as described before [40].

### DNA Manipulations

Transposon mutagenesis and microarray based genetic footprinting were carried out as described before [22]. Chromosomal deletions were created using the previously described method [23] and transferred by generalized transduction with P1 phage as required. *lacZ* reporter strain was generated using the plasmid pCE37 [41]. The over-expression plasmid library construct was a kind gift of Joseph Sklar. Each plasmid contained a 1–3 kb long fragment of *E. coli* MC4100 genome (average fragment size of 2 kb) cloned into the β-lactamase coding sequence of the pBR322 vector. The plasmid pool was electroporated into *E. coli* MG1655 *ΔrfaY* cells, leading to ∼2.5×10^5^ independent over-expressing mutants. For PCR amplification of DNA constructs for cloning, Pfu Ultra polymerase (http://www.stratagene.com) was used, whereas ExTaq DNA polymerase (http://www.takara-bio.com) was used for all other PCR reactions. Restriction endonucleases and T4 ligase were obtained from New England Biolabs (http://www.neb.com). DNA purification kits were obtained from QIAGEN (http://www1.qiagen.com). Primer sequences are available upon request.

### LPS Extraction and Analysis

Structural analysis of LPS samples was performed as described before [42]. LPS was purified from cell lysates after phenol-ether extraction, separated on 14% tricine-SDS-PAGE gel and visualized after silver staining.

### Microscopy

All fluorescence and light microscopy experiments were performed using Zeiss AxioVision 4.5. Time-lapse microscopy was performed in the FC81 flow cell apparatus from Biosurface Technologies Corp (in the absence of flow). SEM analysis was performed using a Philips XL30 Field Emission Scanning Electron Microscope.

### Determination of Hexosamine Content in the Spent Media

For the sake of consistency, a large batch of *ΔcsrA* spent media, grown in LB, was prepared and used as the source of sPNAG for all the experiments throughout this study. The amount of sPNAG present in this stock solution, was estimated by measuring its hexosamine content using 3-methyl-2-benzothiazolone hydrazone hydrochloride (MBTH) method [43] and found to be ∼0.175 mg equivalent of hexosamine per ml of saturated *ΔcsrA* culture supernatant. The quantity of sPNAG in each milliliter of this stock solution was defined to be 1 arbitrary unit (U). Control experiments were also carried out using sPNAG from different preparations of *ΔcsrA* spent media and the results were reproducible.

### sPNAG Purification and Mass Spectrometry

Spent media from 1 liter of saturated *ΔcsrA* culture in LB was passed through a 0.22 µ filter (http://www.nalgene.com/). The cell-free supernatant was concentrated 200 fold by Centriplus YM-100 columns (http://www.millipore.com). The concentrated sample was treated with DNase I (2 mg), RNase A (10 mg), and α-amylase (20 mg) and incubated for 2 hours at room temperature followed by 2 hours at 37°C. Next, sample was treated with Proteinase K (20 mg) for 1 hour at 37°C and 1 hour at 55°C. Enzymes and other proteins were removed by pre-warmed (55°C) phenol: ether extraction, followed by ethanol precipitation. The precipitate was re-suspended in water and fractionated on a S-300 Sephacryl column. The fractions with biofilm-inducing activity were pooled together and concentrated by Centriplus YM-100 columns. The sample was treated with Dispersin B (20 µg) for 4 hours at 37°C. After removing the enzyme by phenol: chloroform extraction, the sample was dialyzed with Spectra/Por cellulose ester membranes with MWCO = 500 (http://www.SPECTRUMLABS.com/) to remove the salt present in Dispersin buffer. Finally, the sample was analyzed by an ESI-LTQ Orbitrap Hybrid mass spectrometer from Thermo Fisher Scientific.

### sPNAG-Induced Biofilm Formation Assay

For all biofilm formation assays, cells and sPNAG were added to fresh LB in such a way as to obtain ∼5×10^8^ cells and 0.1 U of sPNAG per milliliter of the mixture. Biofilm structures were studied or transferred (in case of serial enrichments) 12 hours after exposure to sPNAG at 25°C.

### Preparation of Over-Expression Library Sample for Microarray Hybridization

Plasmids from ∼10^9^ cells of the enriched population (or in case of references, from the maximally diverse unselected library) were extracted using QIAGEN plasmid miniprep kit. The extracted plasmid pool was amplified with two separate PCR reactions, using primer pair pBR_Lib_T7_L and pBR_Lib_R or pBR_Lib_T7_R and pBR_Lib_L. The sequences of these primers are as follows:

pBR_Lib_T7_L: 5′-GTCAACCTGGCTTATCGAAATTAATACGACTCACTATAGGGCTCTTACTGTCATGCCATCCGTA-3′
pBR_Lib_R: 5′-GTTTTCCAATGATGAGCACTTTTA-3′
pBR_Lib_T7_R: 5′-GTCAACCTGGCTTATCGAAATTAATACGACTCACTATAGGGGTTTTCCAATGATGAGCACTTTTA-3′
pBR_Lib_L: 5′-ATAATTCTCTTACTGTCATGCCATCCGTA-3′

The incorporated T7 promoter in pBR_Lib_T7_L and pBR_Lib_T7_R is underlined. Cycling conditions for PCR were 94°C for 2 min; 30 cycles of 94°C for 30 s, 55°C for 30 s, and 72°C for 5 min; and 72°C for 10 min, using ExTaq DNA polymerase. The PCR products from these two reactions were pooled together. The T7 promoter incorporated into the pBR_Lib_T7_L and pBR_Lib_T7_R primer sequences was used to generate RNA from the pooled PCR product in an *in vitro* transcription reaction using Ambion MEGAscript T7 Kit (http://www.ambion.com). Finally the RNA from the previous reaction was reverse transcribed to cDNA, using Cy3-labeled nucleotides with Invitorgen SupreScript II Reverse Transcriptase (http://www.invitrogen.com). The fluorescently labeled cDNA was used for microarray hybridization versus Cy5-labeled fragmented (nebulized) MG1655 genomic DNA. The hybridization data of the two maximally diverse unselected libraries were used as the reference.

### Mapping Spontaneous Mutations Abolishing PNAG Production

Production of PNAG in *S. epidermidis* could be discriminated on congo red indicator plates [44]. In order to extend this to *E. coli*, congo red indicator plates (3% tryptic soy broth, 1% glucose, 0.08% congo red, and 1.5% agar) were supplemented with 200 mM NaCl which was reported to enhance *pga* locus transcription in *E. coli* [16]. Different dilutions of stationary phase cultures of *ΔcsrA* mutants were plated on these plates and incubated for two days at 37°C. PNAG-producing *ΔcsrA* cells formed dark brown colonies whereas *ΔcsrA* mutants defective in producing PNAG were red. To map the location of the mutation which abolished PNAG production, red colonies were picked individually and transduced with a P1 phage lysate obtained from the maximally diverse transposon insertion library. Kanamycin resistant transductants which recovered their PNAG production were identified by replica plating on congo red plates. Dark colonies were picked, and the location of transposon insertion, which should have been linked to the mutation site, was mapped using the same footprinting strategy. The exact location of the mutation was determined after PCR amplification of the candidate genomic locations and subsequent sequencing of the PCR product.

## Supporting Information

Dataset S1 Microarray data for identification of transposon insertion mutants defective in responding to sPNAG. The z-score is calculated based on the average microarray signal of two independent replicates normalized versus five hybridizations of the maximally diverse unselected library. First, the average hybridization intensity value of every ORF from duplicate selection experiments (x) is normalized using mean (<x>) and standard deviation (std(x)) of five hybridizations of the unselected library, representing the null distribution for that ORF. The z-score is calculated using the following equation: z = (x−<x>)/std(x). Therefore, the z-score value for each ORF reflects the relative abundance of transposon insertion events within that ORF or in its vicinity in the enriched population versus the unselected library. More detailed information on hybridization values of each replicate and unselected library is provided in a separate worksheet.
(1.78 MB XLS)

Dataset S2 Microarray data for identification of transposon insertion mutants suppressing *rfaY* deletion phenotype. The z-score is calculated based on the average microarray signal of two independent replicates as explained before (Dataset S1). More detailed information on hybridization values of each replicate and unselected library is provided in a separate worksheet.
(1.94 MB XLS)

Dataset S3 Microarray data for identification of over-expressed fragments suppressing *rfaY* deletion phenotype. The normalized score is calculated by subtraction of the average microarray signal of two maximally diverse unselected libraries from that of two experimental replicates. More detailed information on hybridization values of each replicate and unselected library is provided in a separate worksheet.
(1.58 MB XLS)

Figure S1 Reporter assay for *pga* operon activity in the presence or absence of *csrA* deletion. Up-regulation of the *pga* locus transcription in the *ΔcsrA* background was confirmed by a reporter assay. *pga* promoter was placed upstream of a GFP-coding sequence on a multi-copy plasmid (pPGA'-GFP). The reporter plasmid was electroporated into both wild-type and *ΔcsrA* strains. As shown here, *ΔcsrA* cells strongly express *gfp* while wild-type cells do not show detectable fluorescence. Scale bars, shown in red, correspond to 2 µm.
(0.09 MB PDF)

Figure S2 Comparative ESI LTQ OrbiTrap mass spectrum of Dispersin B-digested PNAG isolated from *E. coli* and *S. aureus*, acquired in positive mode. PNAG sample from *E. coli* was isolated and purified as described in the Materials and Methods section. *S. aureus* PNAG which was isolated from strain MN8m ([1] in Text S1) was a kind gift from Gerald Pier. Purified polysaccharide samples were treated individually with Dispersin B for 1 hour at 37°C and passed through Centriplus YM-10 columns. The flow-through was analyzed by an ESI-LTQ Orbitrap Hybrid mass spectrometer from Thermo Fisher Scientific. The mass spectrums show abundance of different molecular ions (characterized by their *m/z* value) relative to the most abundant ion. As shown here (*E. coli*, panel A, and *S. aureus*, panel B), there are many peaks with identical *m/z* values in both spectra, indicating that the isolated polysaccharides are closely related polymers. A complete list of *m/z* values for all potential mono- and oligosaccharides species that could be generated from an incomplete digestion of a PNAG sample with all possible acetylation patterns are also given in Table S1, as a reference. Panel C shows the mass spectrum of undigested *E. coli* PNAG and panel D corresponds to the spectrum of Dispersin B enzyme. As shown in panel C, intact PNAG can not be analyzed efficiently by OrbiTrap mass spectrum due to its large molecular weight. Therefore, the intensity of peaks acquired for this sample is lower than the noise (e.g., compare the relative intensity of the background peak with *m/z* value of 131.00 in panels C and D). Since digested PNAG samples in panels A and B were passed through a YM-10 filter, those samples should be free from Dispersin B, and spectrum of the enzyme (panel D) was only provided as a control. References in all supporting figures and tables can be found in Text S1.
(0.03 MB PDF)

Figure S3 SEM images of wild-type cells treated with sPNAG (400,000×, 200,000×, and 100,000×, respectively). 50 ml of fresh LB was inoculated with ∼2.5×10^10^ wild-type MG1655 cells with a sPNAG content of 0.1 U per milliliter of the mixture at 25°C. A glass slide was provided as biofilm formation surface in a vertical orientation. The biofilm formed on the surface of the glass slide after 12 hours was fixed with 2.5% glutaraldehyde in 200 mM sodium cadodylate for 60 minutes. Next, it was gently washed twice with 200 mM sodium cadodylate. The sample was post-fixed with 1% osmium tetroxide in sodium cacodylate buffer for 30 minutes. It was washed 4 times, 10 minutes each, with distilled water to remove all the fixative and buffer salts. Next, it was dehydrated sequentially in 35%, 45%, 55%, 65%, 70%, 85%, 95%, and 100% ethanol, 5 minutes each. Then, it was transferred first to 50% ethanol: 50% TMS (tetramethylsilane) followed by 20% ethanol: 80% TMS, 15 minutes each. All the above-mentioned steps were carried out at 4°C. The sample was allowed to air dry at room temperature, and coated with palladium/gold. Finally, the biofilm structures were visualized by a Philips XL30 Field Emission Scanning Electron Microscope.
(0.20 MB PDF)

Figure S4 SDS-PAGE-based analysis of sPNAG-LPS interaction. LPS samples were extracted from wild-type and *ΔrfaY* cells previously treated with sPNAG (1 U/ml) for 12 hours or nothing (as control). The samples were separated on a 14% SDS gel and silver stained. As shown here, sPNAG pre-treatment of the cells did not cause any change in the migration of their LPS samples on SDS-PAGE gel. This could be due to multiple reasons, including the large molecular weight of sPNAG, which is out of the resolution-range of the gel, presence of SDS in the gel and buffer which interferes with electrostatic interactions, or requirement for other components for establishment of the interactions which are present *in vivo*. These results do not rule out the possibility of electrostatic interactions between sPNAG and LPS.
(0.04 MB PDF)

Figure S5 Distribution of acid stress genes in *ΔrfaY* over-expression library. Distribution of acid stress response genes in the microarray result of the *ΔrfaY* over-expression library. Sorted microarray data is divided into 10 equally populated bins and number of genes belonging to “Acid Stress Response Up-regulated” category in each bin was counted and used to calculate hypergeometric p-value to represent the statistical significance of over-representation of those genes in that bin. The bins are pseudo-colored based on the −log_10_(p-value), with yellow color indicating over-representation. Generation of a similar plot has been explained in more detail in Figure 6A.
(0.06 MB PDF)

Figure S6 O-antigen profiling of clinical and laboratory strains of *E. coli*. LPS samples extracted from 11 clinical isolates of *E. coli* together with two samples from the MG1655 strain with or without plasmid pMF19 are analyzed on SDS gels after silver staining. Plasmid pMF19 contains a functional copy of rhamnosyl-transferase gene, *rfaL*, which is mutated in *E. coli* K-12 ([2] in Text S1). Transformation of MG1655 strain with this plasmid allows production of O16 antigen ([3] in Text S1). Among the 11 clinical strains, there were 7 urinary tract infection (UTI) isolates, including UTI-E, -G, -H, -J, -P, -R, and -U, and 4 blood isolates from patients in neonatal intensive care units (NICU), including NICU-2, -4, -10, and -12 ([4] in Text S1). Based on the gel, UTI-U was clearly O-antigen^−^ and UTI-P showed only the first smooth LPS band together with the lipid A-core band. Mutants with LPS banding pattern similar to UTI-P were reported not to react with anti-O antiserum and are considered phenotypically O-antigen^−^ ([5] in Text S1). UTI-E and UTI-G produced less of high molecular weight O-antigen variants, but they were clearly O-antigen^+^ when the gel was overloaded (data not shown).
(0.05 MB PDF)

Figure S7 Horizontal gene transfer rate in sPNAG-based biofilms. Some microbial biofilms promote the rate of horizontal gene transfer, facilitating the spread of antibiotic resistance genes ([6] in Text S1). An equal number (∼10^8^) of donor (tetracycline resistant) and recipient (chloramphenicol resistant) *E. coli* cells were mixed together in LB in the presence or absence of 0.1 U/ml of sPNAG. After 12 hours, serial dilutions of each sample were plated on LB+tetracycline+chloramphenicol plates for CFU counting of conjugants. For each case, results from ten independent samples were averaged and reported. Error bars indicate the standard error of the mean calculated from all measurements. As shown, no significant correlation exists between the presence/absence of sPNAG-based biofilm and the rate of horizontal gene transfer.
(0.03 MB PDF)

Figure S8 Congo red indicator plates for discrimination of sPNAG production in *E. coli*. sPNAG-producing *ΔcsrA* cells (dark brown colonies, left) can be discriminated from otherwise isogenic *ΔcsrA* mutants defective in producing sPNAG (red colonies, right) based on their colony color. Production of PNAG in *S. epidermidis* can be distinguished on congo red indicator plates ([7] in Text S1). In order to extend this to *E. coli*, congo red indicator plates were supplemented with salt, which was reported to enhance *pga* locus transcription in *E. coli* ([8] in Text S1). As shown here, on the indicator plate, sPNAG-producing *ΔcsrA* cells formed dark brown colonies whereas *ΔcsrA pgaA::kan* cells (which had lost their ability to produce sPNAG) were red.
(0.06 MB PDF)

Figure S9 Identification of mutations that abolished sPNAG production in the *ΔcsrA* background. In *S. epidermidis*, PNAG production is subject to a phase-variable regulatory mechanism ([9] in Text S1). In order to understand whether production of sPNAG in *E. coli* undergoes phase variation as well, three mutants were isolated from independent *ΔcsrA* cultures which had lost their sPNAG production ability based on their colony color on congo red indicator plates (Figure S8). When transformed with the plasmid pPGA'-GFP, two of them could express reporter *gfp* downstream of the *pga* promoter while the third one could not. Using generalized phage transduction, the genomic location of the mutation in these three mutants was mapped. Upon sequencing the candidate genomic locations, the identity of the mutations were found. One mutant (mutant I) had an insertion element (IS1E) in 302^nd^ nucleotide of *pgaC* ORF. In the second mutant (mutant II), the whole divergent intergenic region between *pga* operon and *ycdT* gene together with the first 611 nucleotides of *ycdT* ORF and first 1009 nucleotides of *pgaA* ORF was substituted by IS1E. The third mutant (mutant III) was found to have a deletion spanning the entire *nhaR* ORF together with 28 upstream and 176 downstream nucleotides. *NhaR* is required for PNAG production in *E. coli* as it activates *pga* operon transcription ([8] in Text S1). This explains why mutant III does not fluoresce when transformed by the reporter plasmid pPGA'-GFP. In *S. epidermidis*, phase variation in PNAG production is mostly controlled by insertion and excision of an insertion sequence element in PNAG biosynthetic genes ([9] in Text S1), similar to what is happening in case of mutant I and II. However, no reversion back to the producing state was observed in any of these three mutants. These data suggest that inactivation of PNAG production in the *ΔcsrA* background is presumably due to spontaneous loss of function mutations rather than a programmed phase variation process. The high level of PNAG production in the *ΔcsrA* background imposes a considerable energy burden on the cell, therefore loss of function mutations in PNAG biosynthetic pathway may be strongly selected for in this background.
(0.08 MB PDF)

Table S1 Expected *m/z* values for all possible Dispersin B digested PNAG fragments on ESI-LTQ Orbitrap Hybrid mass spectrometer. The molecular weights of all potential mono- and oligosaccharides species that could be generated from incomplete digestion of a PNAG sample with all possible acetylation patterns are calculated. The corresponding *m/z* value for all these molecules and their dehydration products are given here. This table provides a complete list of all potential digestion products, however only a subset of them might exist in the sample and would show up on the spectrum.
(0.03 MB XLS)

Table S2 Validation of microarray data for identification of mutants defective in responding to sPNAG. In-frame deletions of the top candidate ORFs (and a few others) were generated and their behavior was studied in the presence of 0.1 U/ml sPNAG. Only deletion of *rfaP*, *rfaQ*, and *rfaY* impaired the biofilm formation ability of the cells. Other abundant transposon insertion events in different locations of the *rfaQ-K* operon presumably caused their phenotype through polar effects or other indirect interferences with proper biological functioning of *rfaP*, *rfaQ*, and *rfaY* gene products. *rfaF* and *rfaG* deletion mutants showed spontaneous surface-attachment activity in a sPNAG-independent manner, as well. However, this phenotype was much weaker than the sPNAG-induced behavior and was easily distinguishable from it.
(0.02 MB XLS)

Table S3 Effect of Ca^2+^ concentration on cellular response to sPNAG. Divalent cations are postulated to be involved in maintaining outer membrane stability by cross-linking adjacent LPS molecules through their phosphate groups ([10] in Text S1). Consequently, increasing concentration of divalent cations should mask the accessible phosphate groups exposed on the LPS structure. Therefore, we reasoned that increasing concentrations of divalent cations should interfere with any presumable electrostatic interaction between phosphate groups and sPNAG. To test this, wild-type cells were exposed to sPNAG in LB supplemented with different concentrations of Ca^2+^. As shown here, response to sPNAG is lost in Ca^2+^ concentrations higher than 100 µM.
(0.03 MB DOC)

Table S4 Validation of microarray data for identification of suppressor mutations reverting the phenotype of *ΔrfaY* cells. In-frame deletions of the top candidate ORFs (and a few others) were generated in the *ΔrfaY* background and their behavior was studied in the presence of 0.1 U/ml sPNAG. As shown here, deletion of many of the LPS biosynthetic genes suppressed the *rfaY* deletion phenotype. Based on the microarray results, mutants with transposon insertions within or in the vicinity of *znuA* and *znuC*, subunits of zinc uptake transporter of the inner membrane ([11] in Text S1), were significantly enriched in our selection. However, we did not observe any phenotype upon deletion of any of those genes in the *ΔrfaY* background. We also deleted *zur*, the transcriptional repressor of the *znuBC* and *znuA* transcription units ([11] in Text S1) to explore the possibility of the transposon disrupting the repressor binding site and enhancing the expression of these genes, but no phenotype was observed in that case either. Three of the genes involved in *E. coli* type 1 fimbrium (pilus) biosynthesis, *fimA*, *fimB*, and *fimE*, also acquired high scores in our selection. However, since type 1 fimbrium can enhance nonspecific adhesion to biotic and abiotic surfaces ([12,13] in Text S1), we did not focus on those mutants. In case of *rfbD* and *rfbC* mutants, the suppression effect was stronger on glass surface, compared to plastic.
(0.01 MB XLS)

Table S5 Biological function of the *rfaY* suppressors associated with amino acid decarboxylase systems (acid tolerance system) identified in transposon insertion or over-expression libraries. *E. coli* has evolved multiple acid resistance systems. Two of these systems are based upon decarboxylation of glutamate or arginine amino acids at the expense of a proton, which increases intracellular pH ([14] in Text S1). Deletion of *adiY*, the positive regulator of the arginine decarboxylase system, can suppress the *rfaY* deletion phenotype. On the other hand, over-expression of *argR*, the repressor of arginine biosynthesis, reverts the *ΔrfaY* phenotype as well, and arginine is required for maintaining activity of arginine decarboxylase system. These data suggest that inactivation of the arginine decarboxylase system positively contributes to sPNAG-based biofilm formation in the *ΔrfaY* background. The same reasoning can be extended to *gadWYX* over-expression. *gadW* and *gadX* are dual regulators of glutamate decarboxylase system. Depletion of the genes belonging to “Acid Stress Response Up-regulated” category in the microarray results of the over-expression library (Figure S5) suggests that *gadWYX* over-expression exerts its suppressor effect through down-regulation of acid resistance, or more specifically glutamate decarboxylase system. This is in agreement with the predicted behavior of arginine decarboxylase system as well. There is also previous evidence that *gadE*, the transcriptional activator of the acid resistance system in *E. coli*, can up-regulate transcription of the *rfaQ-K* operon ([15] in Text S1). We have also seen that transcription of the *rfaQ-K* operon is decreased upon over-expression of *gadXYW* (Figure 6C), suggesting that *gadXYW* over-expression indirectly contributes to biofilm formation in the *ΔrfaY* background through down-regulation of the acid tolerance system. This presumably happens through structural modifications imposed on LPS.
(0.04 MB DOC)

Table S6 Surface antigen profile of clinical isolates of *E. coli* used in this study. Eleven clinical isolates of *E. coli* were analyzed for presence or absence of O-antigen and K1 capsule along with their ability to respond to sPNAG. Among these 11 clinical strains were 7 urinary tract infection (UTI) isolates, including UTI-E, -G, -H, -J, -P, -R, and -U, and 4 blood isolates from patients in neonatal intensive care units (NICU), including NICU-2, -4, -10, and -12. Presence or absence of O-antigen was determined after running the LPS samples on SDS-PAGE gel and silver staining (Figure S6). K1 capsule production was reported either by sensitivity to bacteriophage E ([16] in Text S1) or based on previous data ([4] in Text S1). NICU-2 was E-phage resistant, suggesting that it is either producing a different variant of K1 capsule or has completely lost its ability to produce K1 capsule due to some spontaneous mutation. Endogenous PNAG production was reported based on previous data ([4] in Text S1). All these strains, except for UTI-H, were capable of expressing the *pga* locus, with UTI-E having relatively weaker expression. Among these strains, 7 formed biofilms in the presence of sPNAG. Two of the UTI isolates (UTI-U and UTI-P), both capable of responding to sPNAG, were O-antigen^−^. Among the O-antigen^+^ strains, there was no significant correlation between the abundance or length of high molecular weight versions of O-antigen and the ability to respond to sPNAG. Among NICU isolates, three K1^+^ NICU isolates were also capable of responding to sPNAG.
(0.03 MB DOC)

Table S7 Bacterial strains used in this study. *: *csrA* has been reported to be conditionally essential in MG1655 ([17] in Text S1) and *csrA* deletion mutants of MG1655 were reported to be capable of growth on LB only after inactivation of the *glgCAP* operon. However, we managed to delete the entire *csrA* ORF in our strain. This discrepancy could be explained either by the differences that might exist between the genetic backgrounds of the strains (which could potentially make *csrA* deletion permissive in one) or acquisition of suppressor mutations that allowed *csrA* deletion. However, we found the growth rate of the mutant to be significantly slower than the wild-type strain.
(0.05 MB XLS)

Table S8 Plasmids and bacteriophages used in this study.
(0.02 MB XLS)

Text S1 References in supporting information.
(0.03 MB DOC)

Video S1 Time-lapse microscopy of wild-type MG1655 cells in presence of 0.1 U/ml of sPNAG in LB. Images were acquired every 30 seconds for the first three hours of exposure to sPNAG.
(4.4 MB WMV)

Video S2 Time-lapse microscopy of wild-type MG1655 cells in LB, in the absence of sPNAG. Images were acquired every 2 minutes for the first 6 hours of the experiment.
(2.5 MB WMV)

Video S3 Time-lapse microscopy of UV-irradiated wild-type MG1655 cells in presence of 0.1 U/ml of sPNAG in LB. Images were acquired every 2 minutes for the first 6 hours of exposure to sPNAG. The viable count of the cells after UV irradiation was found to be zero.
(8.8 MB AVI)
