# Supplementary material for: Genetic Dissection of an Exogenously Induced Biofilm in Laboratory and Clinical Isolates of E. coli
Source: PLoS Pathog. 2009 May 15;5(5):e1000432. doi: 10.1371/journal.ppat.1000432 (PMC2675270; doi:10.1371/journal.ppat.1000432)
Supplement: Figure S1 — Reporter assay for pga operon activity in the presence or absence of csrA deletion. Up-regulation of the pga locus transcription in the ΔcsrA background was confirmed by a reporter assay. pga promoter was placed upstream of a GFP-coding sequence on a multi-copy plasmid (pPGA'-GFP). The reporter plasmid was electroporated into both wild-type and ΔcsrA strains. As shown here, ΔcsrA cells strongly express gfp while wild-type cells do not show detectable fluorescence. Scale bars, shown in red, correspond to 2 µm. (0.09 MB PDF) [file ppat.1000432.s004.pdf]

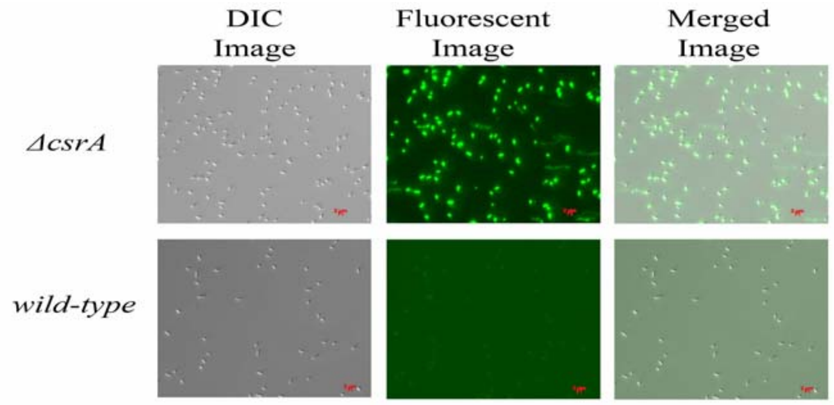

Figure S1. Reporter assay for *pga* operon activity in the presence or absence of *csrA* deletion.

Up-regulation of the *pga* locus transcription in the *ΔcsrA* background was confirmed by a reporter assay. *pga* promoter was placed upstream of a GFP-coding sequence on a multi-copy plasmid (pPGA'-GFP). The reporter plasmid was electroporated into both wild-type and *ΔcsrA* strains. As shown here, *ΔcsrA* cells strongly express *gfp* while wild-type cells do not show detectable fluorescence. Scale bars, shown in red, correspond to 2μm.
