# Supplementary material for: Genetic Dissection of an Exogenously Induced Biofilm in Laboratory and Clinical Isolates of E. coli
Source: PLoS Pathog. 2009 May 15;5(5):e1000432. doi: 10.1371/journal.ppat.1000432 (PMC2675270; doi:10.1371/journal.ppat.1000432)
Supplement: Figure S2 — Comparative ESI LTQ OrbiTrap mass spectrum of Dispersin B-digested PNAG isolated from E. coli and S. aureus, acquired in positive mode. PNAG sample from E. coli was isolated and purified as described in the Materials and Methods section. S. aureus PNAG which was isolated from strain MN8m ([1] in Text S1) was a kind gift from Gerald Pier. Purified polysaccharide samples were treated individually with Dispersin B for 1 hour at 37°C and passed through Centriplus YM-10 columns. The flow-through was analyzed by an ESI-LTQ Orbitrap Hybrid mass spectrometer from Thermo Fisher Scientific. The mass spectrums show abundance of different molecular ions (characterized by their m/z value) relative to the most abundant ion. As shown here (E. coli, panel A, and S. aureus, panel B), there are many peaks with identical m/z values in both spectra, indicating that the isolated polysaccharides are closely related polymers. A complete list of m/z values for all potential mono- and oligosaccharides species that could be generated from an incomplete digestion of a PNAG sample with all possible acetylation patterns are also given in Table S1, as a reference. Panel C shows the mass spectrum of undigested E. coli PNAG and panel D corresponds to the spectrum of Dispersin B enzyme. As shown in panel C, intact PNAG can not be analyzed efficiently by OrbiTrap mass spectrum due to its large molecular weight. Therefore, the intensity of peaks acquired for this sample is lower than the noise (e.g., compare the relative intensity of the background peak with m/z value of 131.00 in panels C and D). Since digested PNAG samples in panels A and B were passed through a YM-10 filter, those samples should be free from Dispersin B, and spectrum of the enzyme (panel D) was only provided as a control. References in all supporting figures and tables can be found in Text S1. (0.03 MB PDF) [file ppat.1000432.s005.pdf]

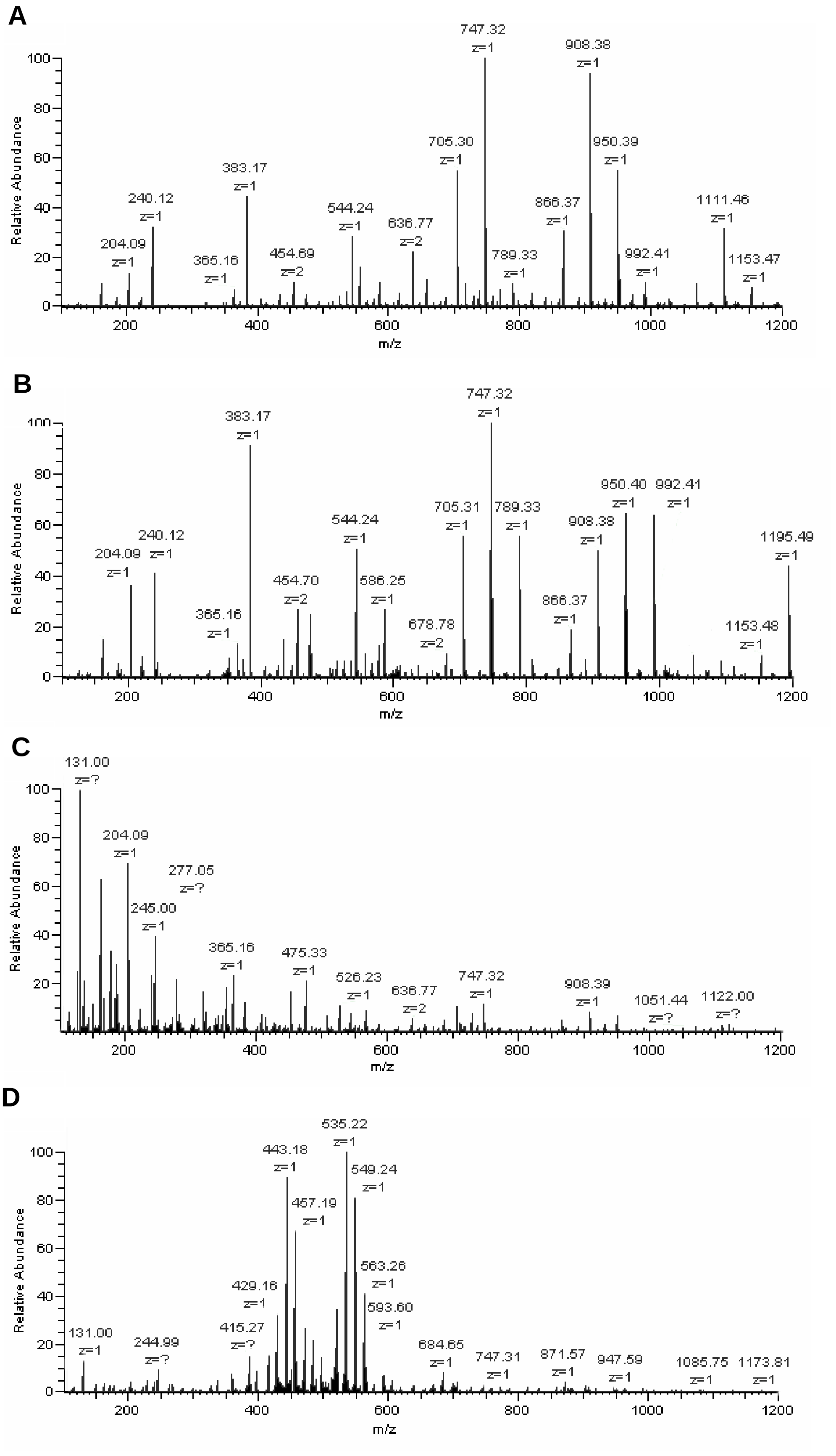
A
747.32 z=1
908.38 z=1
705.30 z=1
950.39 z=1
383.17 z=1
240.12 z=1
544.24 z=1
636.77 z=2
866.37 z=1
1111.46 z=1
204.09 z=1
365.16 z=1
454.69 z=2
789.33 z=1
992.41 z=1
1153.47 z=1
Relative Abundance
m/z
B
747.32 z=1
383.17 z=1
950.40 z=1
992.41 z=1
705.31 z=1
789.33 z=1
544.24 z=1
240.12 z=1
908.38 z=1
1195.49 z=1
204.09 z=1
454.70 z=2
586.25 z=1
866.37 z=1
365.16 z=1
678.78 z=2
1153.48 z=1
Relative Abundance
m/z
C
131.00 z=?
204.09 z=1
277.05 z=?
245.00 z=1
365.16 z=1
475.33 z=1
526.23 z=1
636.77 z=2
747.32 z=1
908.39 z=1
1051.44 z=?
1122.00 z=?
Relative Abundance
m/z
D
535.22 z=1
443.18 z=1
549.24 z=1
457.19 z=1
563.26 z=1
429.16 z=1
593.60 z=1
131.00 z=1
244.99 z=?
415.27 z=?
684.65 z=1
747.31 z=1
871.57 z=1
947.59 z=1
1085.75 z=1
1173.81 z=1
Relative Abundance
m/z

Figure S2. Comparative ESI LTQ OrbiTrap mass spectrum of Dispersin B-digested PNAG isolated from *E. coli* and *S. aureus*, acquired in positive mode.

PNAG sample from *E. coli* was isolated and purified as described in the Materials and Methods section. *S. aureus* PNAG which was isolated from strain MN8m [1] was a kind gift from Gerald Pier. Purified polysaccharide samples were treated individually with Dispersin B for 1 hour at 37ºC and passed through Centriplus YM-10 columns. The flow-through was analyzed by an ESI-LTQ Orbitrap Hybrid mass spectrometer from Thermo Fisher Scientific. The mass spectrums show abundance of different molecular ions (characterized by their *m*/*z* value) relative to the most abundant ion. As shown here (*E. coli*, panel A, and *S. aureus*, panel B), there are many peaks with identical *m/z* values in both spectra, indicating that the isolated polysaccharides are closely related polymers. A complete list of *m/z* values for all potential mono- and oligosaccharides species that could be generated from an incomplete digestion of a PNAG sample with all possible acetylation patterns are also given in Table S1, as a reference. Panel C shows the mass spectrum of undigested *E. coli* PNAG and panel D corresponds to the spectrum of Dispersin B enzyme. As shown in panel C, intact PNAG can not be analyzed efficiently by OrbiTrap mass spectrum due to its large molecular weight. Therefore, the intensity of peaks acquired for this sample is lower than the noise (e.g. compare the relative intensity of the background peak with *m*/*z* value of 131.00 in panel C and D). Since digested PNAG samples in panels A and B were passed through a YM-10 filter, those samples should be free from Dispersin B, and spectrum of the enzyme (panel D) was only provided as a control. References to all supporting figures and tables can be found in file Text S1.
