# Supplementary material for: Genetic Dissection of an Exogenously Induced Biofilm in Laboratory and Clinical Isolates of E. coli
Source: PLoS Pathog. 2009 May 15;5(5):e1000432. doi: 10.1371/journal.ppat.1000432 (PMC2675270; doi:10.1371/journal.ppat.1000432)
Supplement: Figure S3 — SEM images of wild-type cells treated with sPNAG (400,000×, 200,000×, and 100,000×, respectively). 50 ml of fresh LB was inoculated with ∼2.5×10^10^ wild-type MG1655 cells with a sPNAG content of 0.1 U per milliliter of the mixture at 25°C. A glass slide was provided as biofilm formation surface in a vertical orientation. The biofilm formed on the surface of the glass slide after 12 hours was fixed with 2.5% glutaraldehyde in 200 mM sodium cadodylate for 60 minutes. Next, it was gently washed twice with 200 mM sodium cadodylate. The sample was post-fixed with 1% osmium tetroxide in sodium cacodylate buffer for 30 minutes. It was washed 4 times, 10 minutes each, with distilled water to remove all the fixative and buffer salts. Next, it was dehydrated sequentially in 35%, 45%, 55%, 65%, 70%, 85%, 95%, and 100% ethanol, 5 minutes each. Then, it was transferred first to 50% ethanol: 50% TMS (tetramethylsilane) followed by 20% ethanol: 80% TMS, 15 minutes each. All the above-mentioned steps were carried out at 4°C. The sample was allowed to air dry at room temperature, and coated with palladium/gold. Finally, the biofilm structures were visualized by a Philips XL30 Field Emission Scanning Electron Microscope. (0.20 MB PDF) [file ppat.1000432.s006.pdf]

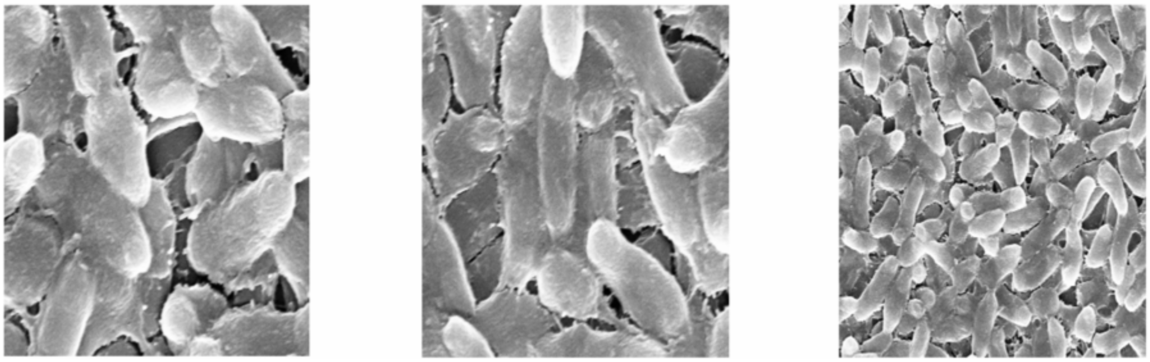

Figure S3. SEM images of wild-type cells treated with sPNAG (400,000x, 200,000x, and 100,000x respectively).

50 ml of fresh LB was inoculated with ~2.5×10^{10} wild-type MG1655 cells with a sPNAG content of 0.1U per milliliter of the mixture at 25ºC. A glass slide was provided as biofilm formation surface in a vertical orientation. The biofilm formed on the surface of the glass slide after 12 hours was fixed with 2.5% glutaraldehyde in 200mM sodium cadodylate for 60 minutes. Next, it was gently washed twice with 200mM sodium cadodylate. The sample was post-fixed with 1% osmium tetroxide in sodium cacodylate buffer for 30 minutes. It was washed 4 times, 10 minutes each, with distilled water to remove all the fixative and buffer salts. Next, it was dehydrated sequentially in 35%, 45%, 55%, 65%, 70%, 85%, 95%, and 100% ethanol, 5 minutes each. Then, it was transferred first to 50% ethanol: 50% TMS (tetramethylsilane) followed by 20% ethanol: 80% TMS, 15 minutes each. All the above mentioned steps were carried out at 4ºC. The sample was allowed to air dry at room temperature, and coated with palladium/gold. Finally, the biofilm structures were visualized by a Philips XL30 Field Emission Scanning Electron Microscope.
