# Supplementary material for: Genetic Dissection of an Exogenously Induced Biofilm in Laboratory and Clinical Isolates of E. coli
Source: PLoS Pathog. 2009 May 15;5(5):e1000432. doi: 10.1371/journal.ppat.1000432 (PMC2675270; doi:10.1371/journal.ppat.1000432)
Supplement: Figure S4 — SDS-PAGE-based analysis of sPNAG-LPS interaction. LPS samples were extracted from wild-type and ΔrfaY cells previously treated with sPNAG (1 U/ml) for 12 hours or nothing (as control). The samples were separated on a 14% SDS gel and silver stained. As shown here, sPNAG pre-treatment of the cells did not cause any change in the migration of their LPS samples on SDS-PAGE gel. This could be due to multiple reasons, including the large molecular weight of sPNAG, which is out of the resolution-range of the gel, presence of SDS in the gel and buffer which interferes with electrostatic interactions, or requirement for other components for establishment of the interactions which are present in vivo. These results do not rule out the possibility of electrostatic interactions between sPNAG and LPS. (0.04 MB PDF) [file ppat.1000432.s007.pdf]

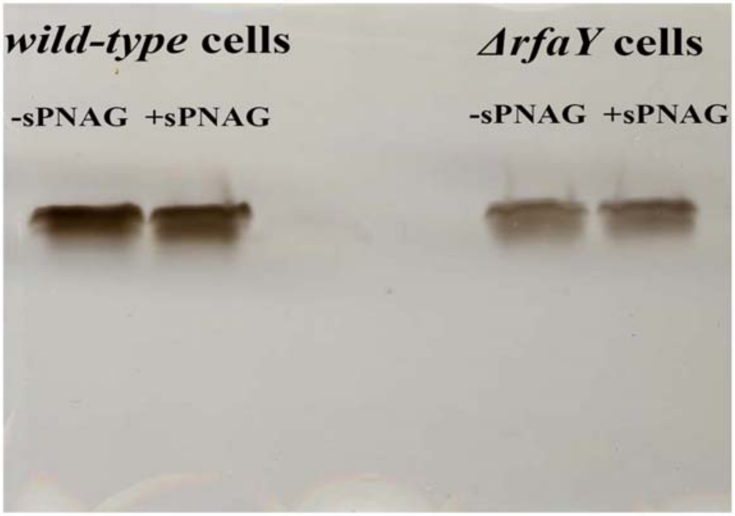

Figure S4. SDS-PAGE-based analysis of sPNAG-LPS interaction.

LPS samples were extracted from wild-type and *ΔrfaY* cells previously treated with sPNAG (1U/ml) for 12 hours or nothing (as control). The samples were separated on a 14% SDS gel and silver stained. As shown here, sPNAG pre-treatment of the cells did not cause any change in the migration of their LPS samples on SDS-PAGE gel. This could be due to multiple reasons, including the large molecular weight of sPNAG which is out of the resolution-range of the gel, presence of SDS in the gel and buffer which interferes with electrostatic interactions, or requirement for other components for establishment of the interactions which are present *in vivo*. These results do not rule out the possibility of electrostatic interactions between sPNAG and LPS.
