# Supplementary material for: Genetic Dissection of an Exogenously Induced Biofilm in Laboratory and Clinical Isolates of E. coli
Source: PLoS Pathog. 2009 May 15;5(5):e1000432. doi: 10.1371/journal.ppat.1000432 (PMC2675270; doi:10.1371/journal.ppat.1000432)
Supplement: Figure S5 — Distribution of acid stress genes in ΔrfaY over-expression library. Distribution of acid stress response genes in the microarray result of the ΔrfaY over-expression library. Sorted microarray data is divided into 10 equally populated bins and number of genes belonging to “Acid Stress Response Up-regulated” category in each bin was counted and used to calculate hypergeometric p-value to represent the statistical significance of over-representation of those genes in that bin. The bins are pseudo-colored based on the −log10(p-value), with yellow color indicating over-representation. Generation of a similar plot has been explained in more detail in Figure 6A. (0.06 MB PDF) [file ppat.1000432.s008.pdf]

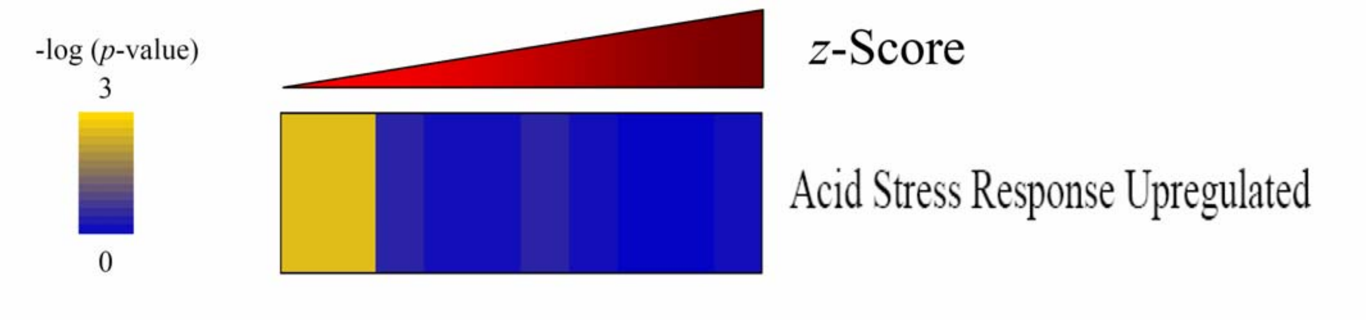

Figure S5. Distribution of acid stress genes in *ΔrfaY* over-expression library.

Distribution of acid stress response genes in the microarray result of the *ΔrfaY* over-expression library. Sorted microarray data is divided into 10 equally populated bins and number of genes belonging to “Acid Stress Response Up-regulated” category in each bin was counted and used to calculate hypergeometric *p*-value to represent the statistical significance of over-representation of those genes in that bin. The bins are pseudo-colored based on the $-\log_{10}(p\text{-value})$, with yellow color indicating over-representation. Generation of a similar plot has been explained in more detailed in Figure 6A.
