# Supplementary material for: Genetic Dissection of an Exogenously Induced Biofilm in Laboratory and Clinical Isolates of E. coli
Source: PLoS Pathog. 2009 May 15;5(5):e1000432. doi: 10.1371/journal.ppat.1000432 (PMC2675270; doi:10.1371/journal.ppat.1000432)
Supplement: Figure S6 — O-antigen profiling of clinical and laboratory strains of E. coli. LPS samples extracted from 11 clinical isolates of E. coli together with two samples from the MG1655 strain with or without plasmid pMF19 are analyzed on SDS gels after silver staining. Plasmid pMF19 contains a functional copy of rhamnosyl-transferase gene, rfaL, which is mutated in E. coli K-12 ([2] in Text S1). Transformation of MG1655 strain with this plasmid allows production of O16 antigen ([3] in Text S1). Among the 11 clinical strains, there were 7 urinary tract infection (UTI) isolates, including UTI-E, -G, -H, -J, -P, -R, and -U, and 4 blood isolates from patients in neonatal intensive care units (NICU), including NICU-2, -4, -10, and -12 ([4] in Text S1). Based on the gel, UTI-U was clearly O-antigen− and UTI-P showed only the first smooth LPS band together with the lipid A-core band. Mutants with LPS banding pattern similar to UTI-P were reported not to react with anti-O antiserum and are considered phenotypically O-antigen− ([5] in Text S1). UTI-E and UTI-G produced less of high molecular weight O-antigen variants, but they were clearly O-antigen+ when the gel was overloaded (data not shown). (0.05 MB PDF) [file ppat.1000432.s009.pdf]

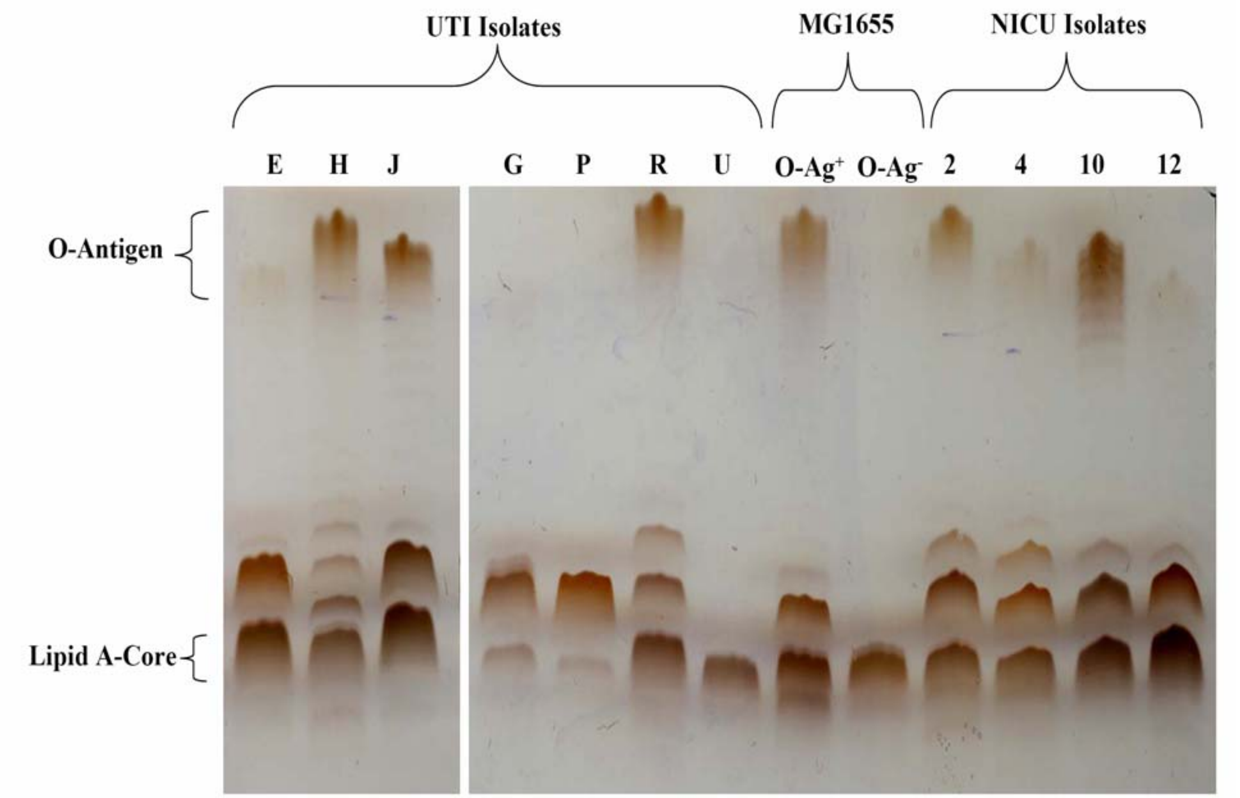

Figure S6. O-antigen profiling of clinical and laboratory strains of *E. coli*.

LPS samples extracted from 11 clinical isolates of *E. coli* together with two samples from the MG1655 strain with or without plasmid pMF19 are analyzed on SDS gels after silver staining. Plasmid pMF19 contains a functional copy of rhamnosyl-transferase gene, *rfaL*, which is mutated in *E. coli* K-12 [2]. Transformation of MG1655 strain with this plasmid allows production of O16 antigen [3]. Among the 11 clinical strains, there were 7 urinary tract infection (UTI) isolates, including UTI-E, -G, -H, -J, -P, -R, and –U, and 4 blood isolates from patients in neonatal intensive care units (NICU), including NICU-2, -4, -10, and -12 [4]. Based on the gel, UTI-U was clearly O-antigen$^-$ and UTI-P showed only the first smooth LPS band together with the lipid A-core band. Mutants with LPS banding pattern similar to UTI-P were reported not to react with anti-O antiserum and are considered phenotypically O-antigen$^-$ [5]. UTI-E and UTI-G produced less of high

molecular weight O-antigen variants, but they were clearly O-antigen^{+} when the gel was overloaded (data not shown).
