# Supplementary material for: Genetic Dissection of an Exogenously Induced Biofilm in Laboratory and Clinical Isolates of E. coli
Source: PLoS Pathog. 2009 May 15;5(5):e1000432. doi: 10.1371/journal.ppat.1000432 (PMC2675270; doi:10.1371/journal.ppat.1000432)
Supplement: Figure S7 — Horizontal gene transfer rate in sPNAG-based biofilms. Some microbial biofilms promote the rate of horizontal gene transfer, facilitating the spread of antibiotic resistance genes ([6] in Text S1). An equal number (∼108) of donor (tetracycline resistant) and recipient (chloramphenicol resistant) E. coli cells were mixed together in LB in the presence or absence of 0.1 U/ml of sPNAG. After 12 hours, serial dilutions of each sample were plated on LB+tetracycline+chloramphenicol plates for CFU counting of conjugants. For each case, results from ten independent samples were averaged and reported. Error bars indicate the standard error of the mean calculated from all measurements. As shown, no significant correlation exists between the presence/absence of sPNAG-based biofilm and the rate of horizontal gene transfer. (0.03 MB PDF) [file ppat.1000432.s010.pdf]

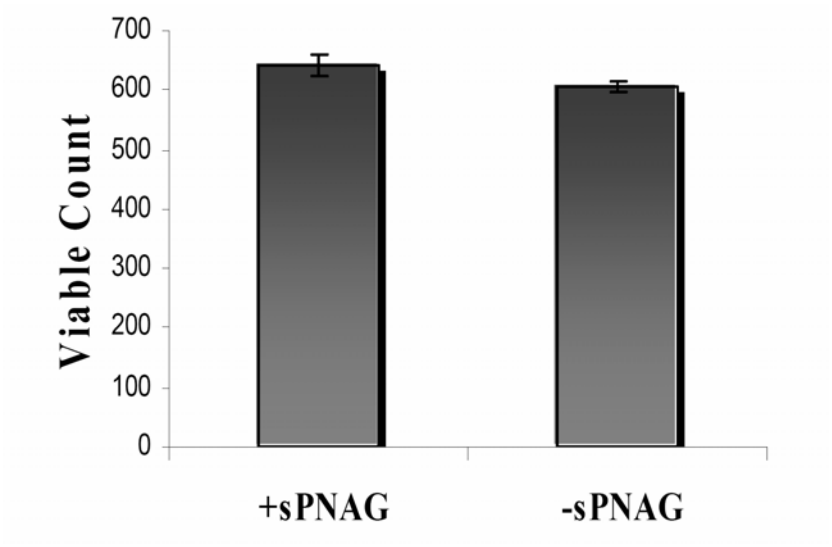

Figure S7. Horizontal gene transfer rate in sPNAG-based biofilms.

Some microbial biofilms promote the rate of horizontal gene transfer, facilitating the spread of antibiotic resistance genes [6]. An equal number (~10^8) of donor (tetracycline resistant) and recipient (chloramphenicol resistant) *E. coli* cells were mixed together in LB in the presence or absence of 0.1U/ml of sPNAG. After 12 hours, serial dilutions of each sample were plated on LB + tetracycline + chloramphenicol plates for CFU counting of conjugants. For each case, results from ten independent samples were averaged and reported. Error bars indicate the standard error of the mean calculated from all measurements. As shown, no significant correlation exists between the presence/absence of sPNAG-based biofilm and the rate of horizontal gene transfer.
