# Supplementary material for: Genetic Dissection of an Exogenously Induced Biofilm in Laboratory and Clinical Isolates of E. coli
Source: PLoS Pathog. 2009 May 15;5(5):e1000432. doi: 10.1371/journal.ppat.1000432 (PMC2675270; doi:10.1371/journal.ppat.1000432)
Supplement: Figure S8 — Congo red indicator plates for discrimination of sPNAG production in E. coli. sPNAG-producing ΔcsrA cells (dark brown colonies, left) can be discriminated from otherwise isogenic ΔcsrA mutants defective in producing sPNAG (red colonies, right) based on their colony color. Production of PNAG in S. epidermidis can be distinguished on congo red indicator plates ([7] in Text S1). In order to extend this to E. coli, congo red indicator plates were supplemented with salt, which was reported to enhance pga locus transcription in E. coli ([8] in Text S1). As shown here, on the indicator plate, sPNAG-producing ΔcsrA cells formed dark brown colonies whereas ΔcsrA pgaA::kan cells (which had lost their ability to produce sPNAG) were red. (0.06 MB PDF) [file ppat.1000432.s011.pdf]

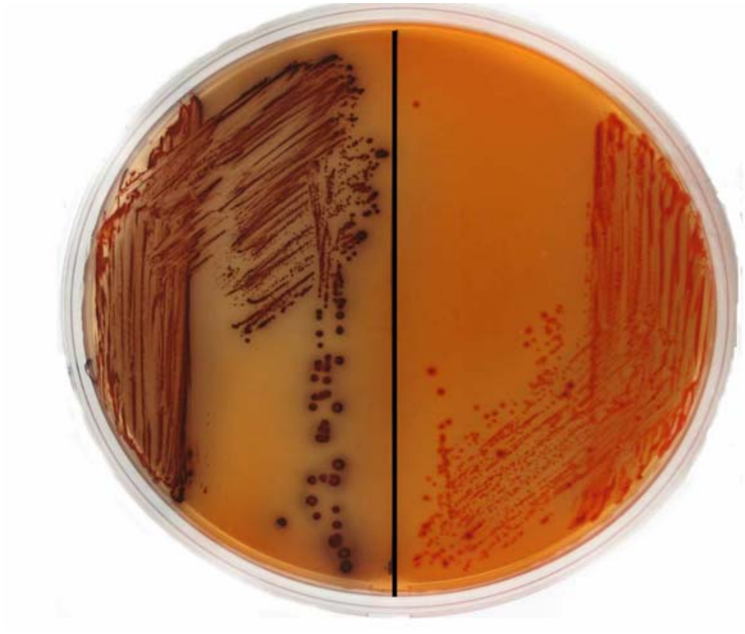

Figure S8. Congo red indicator plates for discrimination of sPNAG production in *E. coli*.

sPNAG-producing *ΔcsrA* cells (dark brown colonies, left) can be discriminated from otherwise isogenic *ΔcsrA* mutants defective in producing sPNAG (red colonies, right) based on their colony color. Production of PNAG in *S. epidermidis* can be distinguished on congo red indicator plates [7]. In order to extend this to *E. coli*, congo red indicator plates were supplemented with salt, which was reported to enhance *pga* locus transcription in *E. coli* [8]. As shown here, on the indicator plate, sPNAG-producing *ΔcsrA* cells formed dark brown colonies whereas *ΔcsrA pgaA::kan* cells (which had lost their ability to produce sPNAG) were red.
