# Supplementary material for: Genetic Dissection of an Exogenously Induced Biofilm in Laboratory and Clinical Isolates of E. coli
Source: PLoS Pathog. 2009 May 15;5(5):e1000432. doi: 10.1371/journal.ppat.1000432 (PMC2675270; doi:10.1371/journal.ppat.1000432)
Supplement: Figure S9 — Identification of mutations that abolished sPNAG production in the ΔcsrA background. In S. epidermidis, PNAG production is subject to a phase-variable regulatory mechanism ([9] in Text S1). In order to understand whether production of sPNAG in E. coli undergoes phase variation as well, three mutants were isolated from independent ΔcsrA cultures which had lost their sPNAG production ability based on their colony color on congo red indicator plates (Figure S8). When transformed with the plasmid pPGA'-GFP, two of them could express reporter gfp downstream of the pga promoter while the third one could not. Using generalized phage transduction, the genomic location of the mutation in these three mutants was mapped. Upon sequencing the candidate genomic locations, the identity of the mutations were found. One mutant (mutant I) had an insertion element (IS1E) in 302nd nucleotide of pgaC ORF. In the second mutant (mutant II), the whole divergent intergenic region between pga operon and ycdT gene together with the first 611 nucleotides of ycdT ORF and first 1009 nucleotides of pgaA ORF was substituted by IS1E. The third mutant (mutant III) was found to have a deletion spanning the entire nhaR ORF together with 28 upstream and 176 downstream nucleotides. NhaR is required for PNAG production in E. coli as it activates pga operon transcription ([8] in Text S1). This explains why mutant III does not fluoresce when transformed by the reporter plasmid pPGA'-GFP. In S. epidermidis, phase variation in PNAG production is mostly controlled by insertion and excision of an insertion sequence element in PNAG biosynthetic genes ([9] in Text S1), similar to what is happening in case of mutant I and II. However, no reversion back to the producing state was observed in any of these three mutants. These data suggest that inactivation of PNAG production in the ΔcsrA background is presumably due to spontaneous loss of function mutations rather than a programmed phase variation process. The high [file ppat.1000432.s012.pdf]

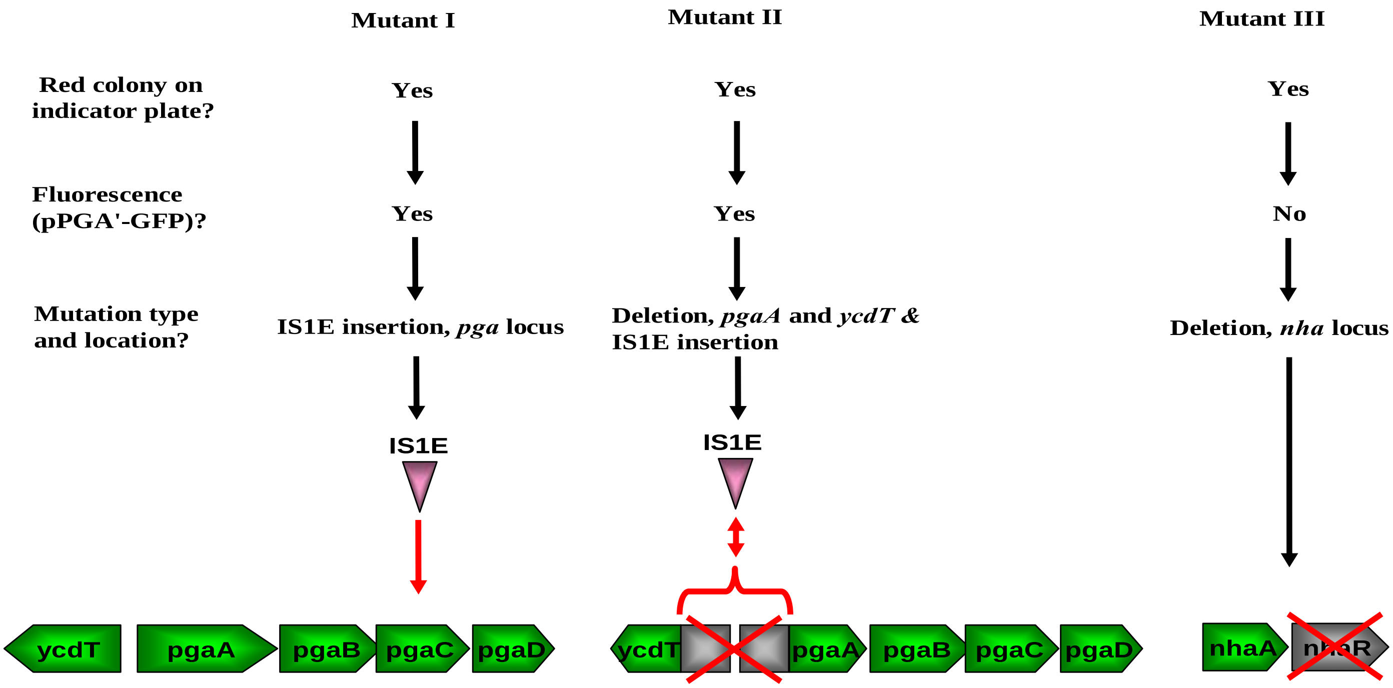

Figure S9. Identification of mutations that abolished sPNAG production in the *ΔcsrA* background.

In *S. epidermidis*, PNAG production is subject to a phase-variable regulatory mechanism [7]. In order to understand whether production of sPNAG in *E. coli* undergoes phase variation as well, three mutants were isolated from independent *ΔcsrA* cultures which had lost their sPNAG production ability based on their colony color on congo red indicator plates (Figure S8). When transformed with the plasmid pPGA'-GFP, two of them could express reporter *gfp* downstream of the *pga* promoter while the third one could not. Using generalized phage transduction, the genomic location of the mutation in these three mutants was mapped. Upon sequencing the candidate genomic locations, the identity of the mutations were found. One mutant (mutant I) had an insertion element (IS1E) in 302nd nucleotide of *pgaC* ORF. In the second mutant (mutant II), the whole divergent intergenic region between *pga* operon and *ycdT* gene together with the first 611 nucleotides of *ycdT* ORF and first 1009 nucleotides of *pgaA* ORF was substituted by IS1E. The third mutant (mutant III) was found to have a deletion spanning the entire *nhaR* ORF together with 28 upstream and 176 downstream nucleotides. *NhaR* is required for PNAG production in *E. coli* as it activates *pga* operon

transcription [8]. This explains why mutant III does not fluoresce when transformed by the reporter plasmid pPGA'-GFP. In *S. epidermidis*, phase variation in PNAG production is mostly controlled by insertion and excision of an insertion sequence element in PNAG biosynthetic genes [7], similar to what is happening in case of mutant I and II. However, no reversion back to the producing state was observed in any of these three mutants. These data suggest that inactivation of PNAG production in the *ΔcsrA* background is presumably due to spontaneous loss of function mutations rather than a programmed phase variation process. The high level of PNAG production in the *ΔcsrA* background imposes a considerable energy burden on the cell, therefore loss of function mutations in PNAG biosynthetic pathway may be strongly selected for in this background.
