# Supplementary material for: Genetic Dissection of an Exogenously Induced Biofilm in Laboratory and Clinical Isolates of E. coli
Source: PLoS Pathog. 2009 May 15;5(5):e1000432. doi: 10.1371/journal.ppat.1000432 (PMC2675270; doi:10.1371/journal.ppat.1000432)
Supplement: Table S3 — Effect of Ca2+ concentration on cellular response to sPNAG. Divalent cations are postulated to be involved in maintaining outer membrane stability by cross-linking adjacent LPS molecules through their phosphate groups ([10] in Text S1). Consequently, increasing concentration of divalent cations should mask the accessible phosphate groups exposed on the LPS structure. Therefore, we reasoned that increasing concentrations of divalent cations should interfere with any presumable electrostatic interaction between phosphate groups and sPNAG. To test this, wild-type cells were exposed to sPNAG in LB supplemented with different concentrations of Ca2+. As shown here, response to sPNAG is lost in Ca2+ concentrations higher than 100 µM. (0.03 MB DOC) [file ppat.1000432.s015.doc]

|  |  | | | | | |
| --- | --- | --- | --- | --- | --- | --- |
| **Ca^2+^ Concentration (M)** | 10^-2^ | 10^-3^ | 10^-4^ | 10^-5^ | 10^-6^ | 10^-7^ |
| **Response to sPNAG** | - | - | + | + | + | + |

**Table S3. Effect of Ca^2+^ concentration on cellular response to sPNAG.**

Divalent cations are postulated to be involved in maintaining outer membrane stability by cross-linking adjacent LPS molecules through their phosphate groups [10]. Consequently, increasing concentration of divalent cations should mask the accessible phosphate groups exposed on the LPS structure. Therefore, we reasoned that increasing concentrations of divalent cations should interfere with any presumable electrostatic interaction between phosphate groups and sPNAG. To test this, wild-type cells were exposed to sPNAG in LB supplemented with different concentrations of Ca^2+^. As shown here, response to sPNAG is lost in Ca^2+^ concentrations higher than 100μM.
