# Supplementary material for: Genetic Dissection of an Exogenously Induced Biofilm in Laboratory and Clinical Isolates of E. coli
Source: PLoS Pathog. 2009 May 15;5(5):e1000432. doi: 10.1371/journal.ppat.1000432 (PMC2675270; doi:10.1371/journal.ppat.1000432)
Supplement: Table S5 — Biological function of the rfaY suppressors associated with amino acid decarboxylase systems (acid tolerance system) identified in transposon insertion or over-expression libraries. E. coli has evolved multiple acid resistance systems. Two of these systems are based upon decarboxylation of glutamate or arginine amino acids at the expense of a proton, which increases intracellular pH ([14] in Text S1). Deletion of adiY, the positive regulator of the arginine decarboxylase system, can suppress the rfaY deletion phenotype. On the other hand, over-expression of argR, the repressor of arginine biosynthesis, reverts the ΔrfaY phenotype as well, and arginine is required for maintaining activity of arginine decarboxylase system. These data suggest that inactivation of the arginine decarboxylase system positively contributes to sPNAG-based biofilm formation in the ΔrfaY background. The same reasoning can be extended to gadWYX over-expression. gadW and gadX are dual regulators of glutamate decarboxylase system. Depletion of the genes belonging to “Acid Stress Response Up-regulated” category in the microarray results of the over-expression library (Figure S5) suggests that gadWYX over-expression exerts its suppressor effect through down-regulation of acid resistance, or more specifically glutamate decarboxylase system. This is in agreement with the predicted behavior of arginine decarboxylase system as well. There is also previous evidence that gadE, the transcriptional activator of the acid resistance system in E. coli, can up-regulate transcription of the rfaQ-K operon ([15] in Text S1). We have also seen that transcription of the rfaQ-K operon is decreased upon over-expression of gadXYW (Figure 6C), suggesting that gadXYW over-expression indirectly contributes to biofilm formation in the ΔrfaY background through down-regulation of the acid tolerance system. This presumably happens through structural modifications imposed on LPS. (0.04 MB DOC) [file ppat.1000432.s017.doc]

|  | **Identified in:** | **Biological Function** |
| --- | --- | --- |
| **Gene** |  |  |
| *adiY* | transposon insertion library | positive regulator of arginine decarboxylase system |
| *argR* | over-expression library | negative regulator of arginine biosynthesis |
| *gadX* | over-expression library | dual regulator of glutamate decarboxylase system |
| *gadW* | over-expression library | dual regulator of glutamate decarboxylase system |

**Table S5.** **Biological function of the *rfaY* suppressors associated with amino acid decarboxylase systems (acid tolerance system) identified in transposon insertion or over-expression libraries.**

*E. coli* has evolved multiple acid resistance systems. Two of these systems are based upon decarboxylation of glutamate or arginine amino acids at the expense of a proton, which increases intracellular pH [14]. Deletion of *adiY*, the positive regulator of the arginine decarboxylase system, can suppress the *rfaY* deletion phenotype. On the other hand, over-expression of *argR*, the repressor of arginine biosynthesis, reverts the *ΔrfaY* phenotype as well, and arginine is required for maintaining activity of arginine decarboxylase system. These data suggest that inactivation of the arginine decarboxylase system positively contributes to sPNAG-based biofilm formation in the *ΔrfaY* background. The same reasoning can be extended to *gadWYX* over-expression. *gadW* and *gadX* are dual regulators of glutamate decarboxylase system. Depletion of the genes belonging to “Acid Stress Response Up-regulated” category in the microarray results of the over-expression library (Figure S5) suggests that *gadWYX* over-expression exerts its suppressor effect through down-regulation of acid resistance, or more specifically glutamate decarboxylase system. This is in agreement with the predicted behavior of arginine decarboxylase system as well.

There is also previous evidence that *gadE*, the transcriptional activator of the acid resistance system in *E. coli*, can up-regulate transcription of the *rfaQ-K* operon [15]. We have also seen that transcription of the *rfaQ-K* operon is decreased upon over-expression of *gadXYW* (Figure 6C), suggesting that *gadXYW* over-expression indirectly contributes to biofilm formation in the *ΔrfaY* background through down-regulation of the acid tolerance system. This presumably happens through structural modifications imposed on LPS.
