# Supplementary material for: Genetic Dissection of an Exogenously Induced Biofilm in Laboratory and Clinical Isolates of E. coli
Source: PLoS Pathog. 2009 May 15;5(5):e1000432. doi: 10.1371/journal.ppat.1000432 (PMC2675270; doi:10.1371/journal.ppat.1000432)
Supplement: Table S6 — Surface antigen profile of clinical isolates of E. coli used in this study. Eleven clinical isolates of E. coli were analyzed for presence or absence of O-antigen and K1 capsule along with their ability to respond to sPNAG. Among these 11 clinical strains were 7 urinary tract infection (UTI) isolates, including UTI-E, -G, -H, -J, -P, -R, and -U, and 4 blood isolates from patients in neonatal intensive care units (NICU), including NICU-2, -4, -10, and -12. Presence or absence of O-antigen was determined after running the LPS samples on SDS-PAGE gel and silver staining (Figure S6). K1 capsule production was reported either by sensitivity to bacteriophage E ([16] in Text S1) or based on previous data ([4] in Text S1). NICU-2 was E-phage resistant, suggesting that it is either producing a different variant of K1 capsule or has completely lost its ability to produce K1 capsule due to some spontaneous mutation. Endogenous PNAG production was reported based on previous data ([4] in Text S1). All these strains, except for UTI-H, were capable of expressing the pga locus, with UTI-E having relatively weaker expression. Among these strains, 7 formed biofilms in the presence of sPNAG. Two of the UTI isolates (UTI-U and UTI-P), both capable of responding to sPNAG, were O-antigen−. Among the O-antigen+ strains, there was no significant correlation between the abundance or length of high molecular weight versions of O-antigen and the ability to respond to sPNAG. Among NICU isolates, three K1+ NICU isolates were also capable of responding to sPNAG. (0.03 MB DOC) [file ppat.1000432.s018.doc]

|  | **UTI Isolates** | | | | | | | **NICU Isolates** | | | |
| --- | --- | --- | --- | --- | --- | --- | --- | --- | --- | --- | --- |
| **Strain ID** | E | G | H | J | P | R | U | 2 | 4 | 10 | 12 |
| **Producing Endogenous PNAG** | +- | + | - | + | + | + | + | + | + | + | + |
| **O-Antigen** | + | + | + | + | - | + | - | + | + | + | + |
| **K1-Capsule** | - | - | - | - | - | - | - | +^*^ | + | + | + |
| **Response to sPNAG** | - | - | + | + | + | - | + | - | + | + | + |

*: Even though reported to be K1^+^, is resistant to K1-specific phage E

**Table S6.** **Surface antigen profile of clinical isolates of *E. coli* used in this study.**

Eleven clinical isolates of *E. coli* were analyzed for presence or absence of O-antigen and K1 capsule along with their ability to respond to sPNAG. Among these 11 clinical strains were 7 urinary tract infection (UTI) isolates, including UTI-E, -G, -H, -J, -P, -R, and –U, and 4 blood isolates from patients in neonatal intensive care units (NICU), including NICU-2, -4, -10, and -12. Presence or absence of O-antigen was determined after running the LPS samples on SDS-PAGE gel and silver staining (Figure S6). K1 capsule production was reported either by sensitivity to bacteriophage E [16] or based on previous data [4]. NICU-2 was E-phage resistant, suggesting that it is either producing a different variant of K1 capsule or has completely lost its ability to produce K1 capsule due to some spontaneous mutation. Endogenous PNAG production was reported based on previous data [4]. All these strains, except for UTI-H, were capable of expressing the *pga* locus, with UTI-E having relatively weaker expression. Among these strains, 7 formed biofilms in the presence of sPNAG. Two of the UTI isolates (UTI-U and UTI-P), both capable of responding to sPNAG, were O-antigen^~~-~~^. Among the O-antigen^+^ strains, there was no significant correlation between the abundance or length of high molecular weight versions of O-antigen and the ability to respond to sPNAG. Among NICU isolates, three K1^+^ NICU isolates were also capable of responding to sPNAG.
