# Supplementary material for: Genetic Dissection of an Exogenously Induced Biofilm in Laboratory and Clinical Isolates of E. coli
Source: PLoS Pathog. 2009 May 15;5(5):e1000432. doi: 10.1371/journal.ppat.1000432 (PMC2675270; doi:10.1371/journal.ppat.1000432)
Supplement: Text S1 — References in supporting information. (0.03 MB DOC) [file ppat.1000432.s021.doc]

**References:**

1. Jefferson KK, Cramton SE, Gotz F, Pier GB (2003) Identification of a 5-nucleotide sequence that controls expression of the ica locus in Staphylococcus aureus and characterization of the DNA-binding properties of IcaR. Mol Microbiol 48: 889-899.

2. Liu D, Reeves PR (1994) Escherichia coli K12 regains its O antigen. Microbiology 140 ( Pt 1): 49-57.

3. Marolda CL, Vicarioli J, Valvano MA (2004) Wzx proteins involved in biosynthesis of O antigen function in association with the first sugar of the O-specific lipopolysaccharide subunit. Microbiology 150: 4095-4105.

4. Cerca N, Maira-Litran T, Jefferson KK, Grout M, Goldmann DA, et al. (2007) Protection against Escherichia coli infection by antibody to the Staphylococcus aureus poly-N-acetylglucosamine surface polysaccharide. Proc Natl Acad Sci U S A 104: 7528-7533.

5. Kusecek B, Wloch H, Mercer A, Vaisanen V, Pluschke G, et al. (1984) Lipopolysaccharide, capsule, and fimbriae as virulence factors among O1, O7, O16, O18, or O75 and K1, K5, or K100 Escherichia coli. Infect Immun 43: 368-379.

6. Fux CA, Costerton JW, Stewart PS, Stoodley P (2005) Survival strategies of infectious biofilms. Trends Microbiol 13: 34-40.

7. Ziebuhr W, Loessner I, Krimmer V, Hacker J (2001) Methods to detect and analyze phenotypic variation in biofilm-forming Staphylococci. Methods Enzymol 336: 195-205.

8. Goller C, Wang X, Itoh Y, Romeo T (2006) The cation-responsive protein NhaR of Escherichia coli activates pgaABCD transcription, required for production of the biofilm adhesin poly-beta-1,6-N-acetyl-D-glucosamine. J Bacteriol 188: 8022-8032.

9. Ziebuhr W, Krimmer V, Rachid S, Lossner I, Gotz F, et al. (1999) A novel mechanism of phase variation of virulence in Staphylococcus epidermidis: evidence for control of the polysaccharide intercellular adhesin synthesis by alternating insertion and excision of the insertion sequence element IS256. Mol Microbiol 32: 345-356.

10. Yethon JA, Heinrichs DE, Monteiro MA, Perry MB, Whitfield C (1998) Involvement of waaY, waaQ, and waaP in the modification of Escherichia coli lipopolysaccharide and their role in the formation of a stable outer membrane. J Biol Chem 273: 26310-26316.

11. Patzer SI, Hantke K (1998) The ZnuABC high-affinity zinc uptake system and its regulator Zur in Escherichia coli. Mol Microbiol 28: 1199-1210.

12. Otto K, Norbeck J, Larsson T, Karlsson KA, Hermansson M (2001) Adhesion of type 1-fimbriated Escherichia coli to abiotic surfaces leads to altered composition of outer membrane proteins. J Bacteriol 183: 2445-2453.

13. Pratt LA, Kolter R (1998) Genetic analysis of Escherichia coli biofilm formation: roles of flagella, motility, chemotaxis and type I pili. Mol Microbiol 30: 285-293.

14. Foster JW (2004) Escherichia coli acid resistance: tales of an amateur acidophile. Nat Rev Microbiol 2: 898-907.

15. Hommais F, Krin E, Coppee JY, Lacroix C, Yeramian E, et al. (2004) GadE (YhiE): a novel activator involved in the response to acid environment in Escherichia coli. Microbiology 150: 61-72.

16. Annunziato PW, Wright LF, Vann WF, Silver RP (1995) Nucleotide sequence and genetic analysis of the neuD and neuB genes in region 2 of the polysialic acid gene cluster of Escherichia coli K1. J Bacteriol 177: 312-319.

17. Timmermans J, Van Melderen L (2009) Conditional essentiality of the csrA gene in Escherichia coli. J Bacteriol 191: 1722-1724.
